# Supplementary material for: Transient and tunable CRISPRa regulation of APOBEC/AID genes for targeting hepatitis B virus
Source: Mol Ther Nucleic Acids. 2023 Apr 20;32:478–93. doi: 10.1016/j.omtn.2023.04.016 (PMC10176074; doi:10.1016/j.omtn.2023.04.016)
Supplement: Document S1. Figures S1–S16 and Table S18 and S19 [file mmc1.pdf]

OMTN, Volume *32*

Supplemental information

Transient and tunable CRISPRa regulation of APOBEC/AID genes for targeting hepatitis B virus

Dmitry Kostyushev, Sergey Brezgin, Anastasiya Kostyusheva, Natalia Ponomareva, Ekaterina Bayurova, Natalia Zakirova, Alla Kondrashova, Irina Goptar, Anastasiya Nikiforova, Anna Sudina, Yurii Babin, Ilya Gordeychuk, Alexander Lukashev, Andrey A. Zamyatnin Jr., Alexander Ivanov, and Vladimir Chulanov

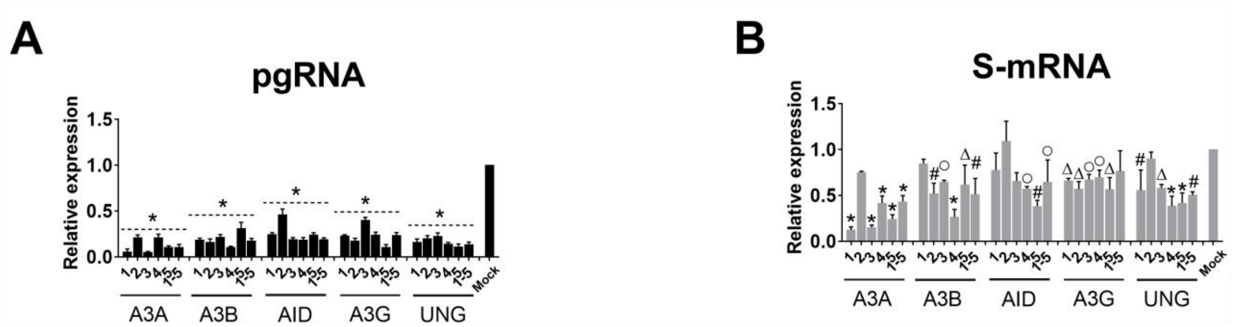

Figure S1. Screening for sgRNAs with highest anti-HBV activity. Anti-HBV activity of sgRNAs targeting A3A, A3B, AID, A3G and UNG genes measured by (A) pgRNA and (B) S-mRNA levels. HBV RNA is provided relative to GAPDH mRNA. $^{\circ}p < 0.05$, $^{\Delta}p < 0.01$, $^{\#}p < 0.001$, $^{*}p < 0.0001$. *Mock* – dCas9-p300 transfected with a non-targeting sgRNA. Numbers indicate individual sgRNAs, 1-5 corresponds to all five sgRNAs targeting a single gene used in combination.

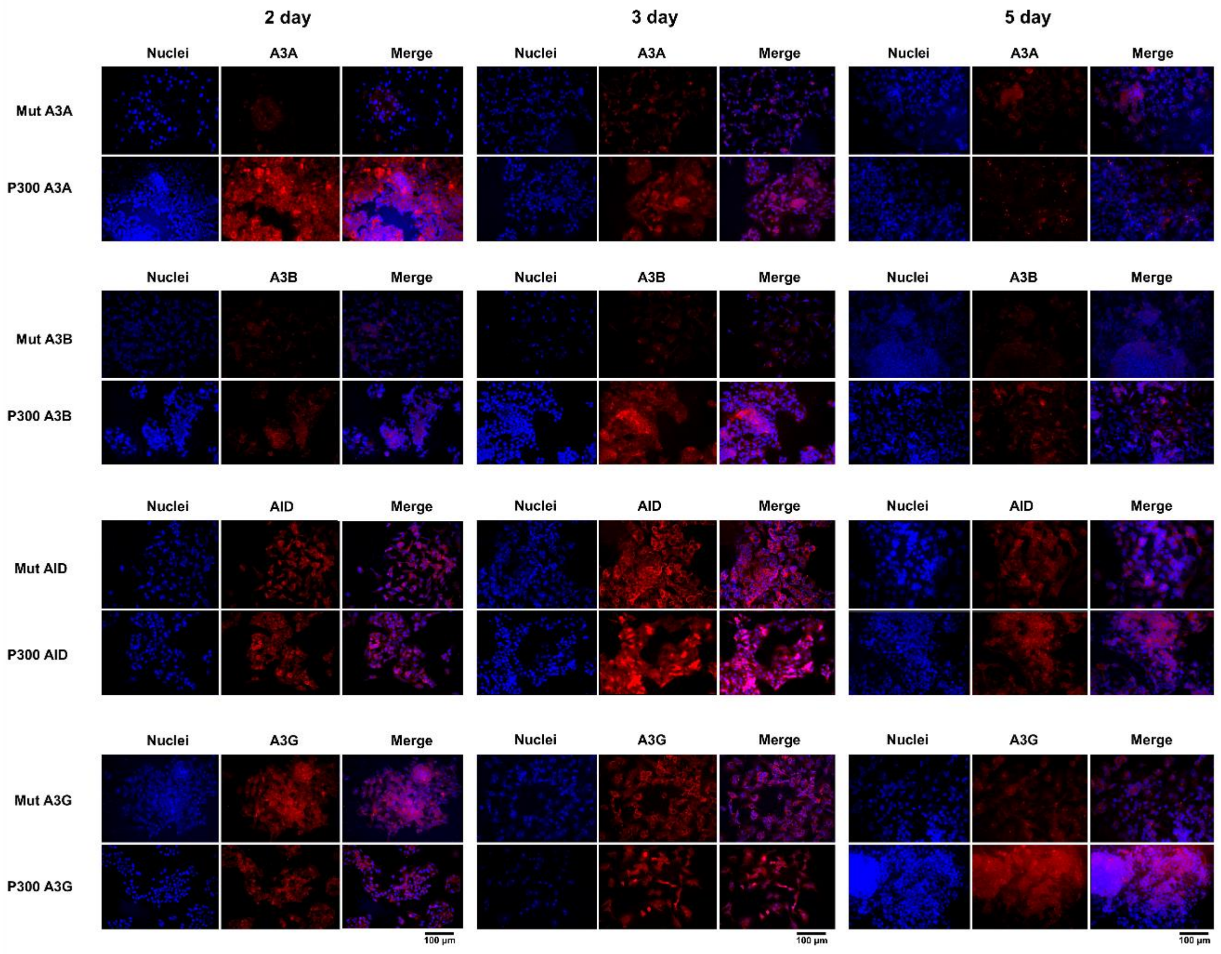

Figure S2. Immunostaining for APOBEC/AID factors. Mut – cells transfected with a mutant form of dCas9-p300 and a targeting sgRNA, P300 – cells transfected with dCas9-p300 and a targeting sgRNA. HepG2 cells were stained for a corresponding APOBEC/AID protein at specified days; cell nuclei are labeled with a Hoechst33342 (blue). Note: these images are used as merged images in Figure 1E.

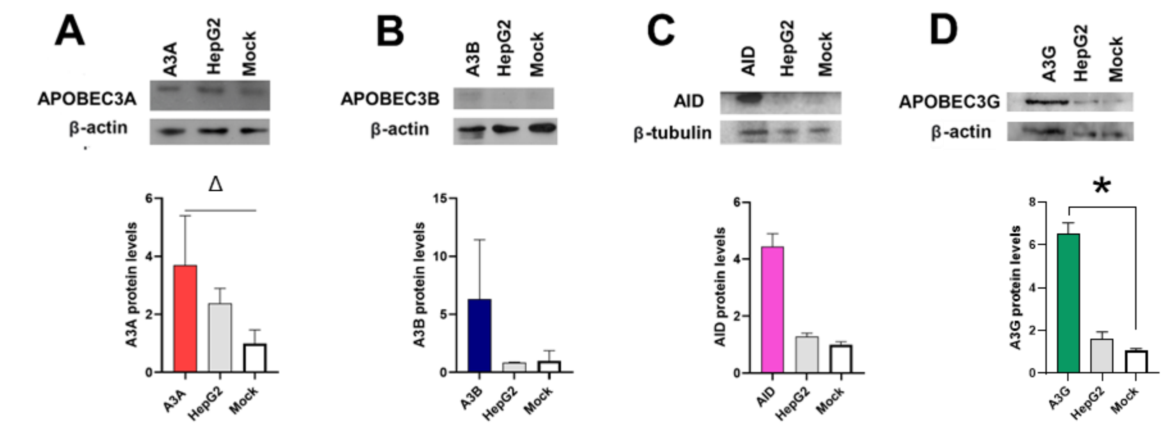

Figure S3. Western blot analysis APOBEC/AID expression. Protein expression of (A) A3A, (B) A3B, (C) AID and (D) A3G. Western blot analysis was performed 42 hours post transfection. Mock, HepG2 cells transfected with dCas9-p300 with a non-targeting sgRNA. Values are expressed as means ± standard deviation of triplicate experiments. HepG2 – not transfected HepG2 cells. $^{\Delta}p < 0.01$, $^{*}p < 0.0001$.

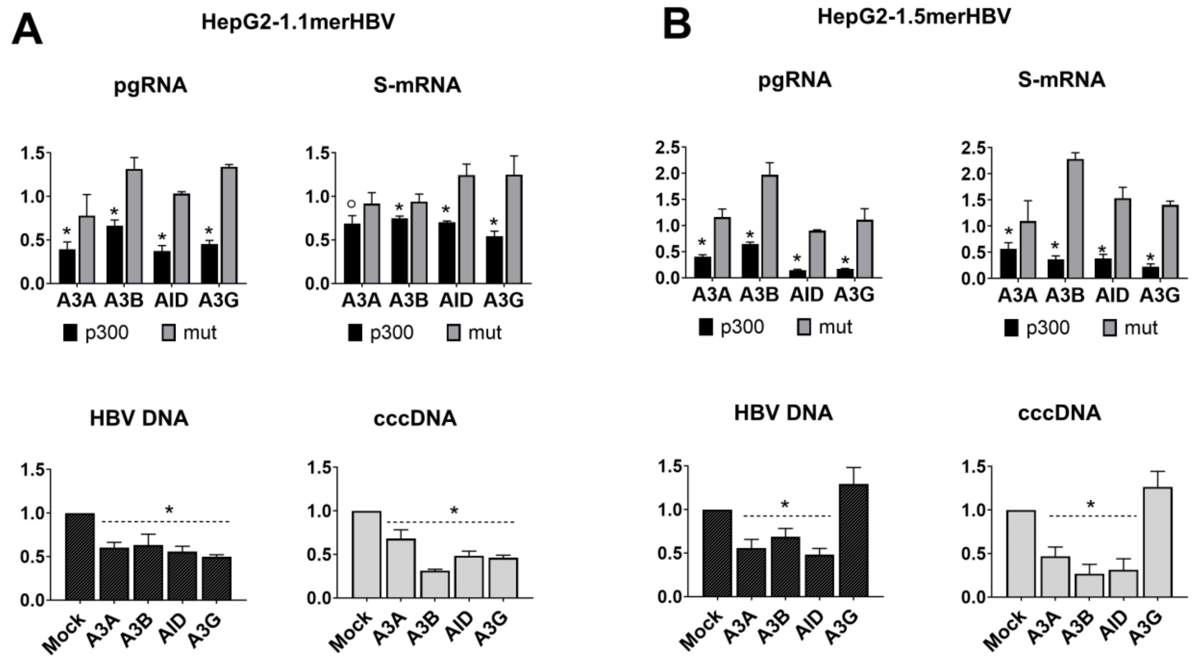

Figure S4. Suppression of HBV replication by CRISPRa at stable cell lines. Anti-HBV activity was analyzed at (A) HepG2-1.1merHBV cells with HBV replication induced from a tet-on CMV promoter de novo by adding doxycycline for 24 hours and (B) HepG2-1.5merHBV cells where HBV is constitutively produced from a wild-type promoter. Anti-HBV activity was measured 5 days post transfection; transfected cells were selected using blasticidin treatment as previously described[1]. HBV DNA and cccDNA are provided relative to genomic β-globin; HBV pgRNA and S-mRNA are relative to GAPDH mRNA. Cell lines were transfected with dCas9-p300 or a mutant form of dCas9-p300 with an sgRNA targeting A3A, A3B, AID or A3G. . $^{\circ}p < 0.05$, $^{\Delta}p < 0.01$, $^{\#}p < 0.001$, $^{*}p < 0.0001$. *Mut* – cells transfected with a mutant form of dCas9-p300 and a

targeting sgRNA, *P300* – cells transfected with dCas9-p300 and a targeting sgRNA. *Mock* – dCas9-p300 transfected with a non-targeting sgRNA.

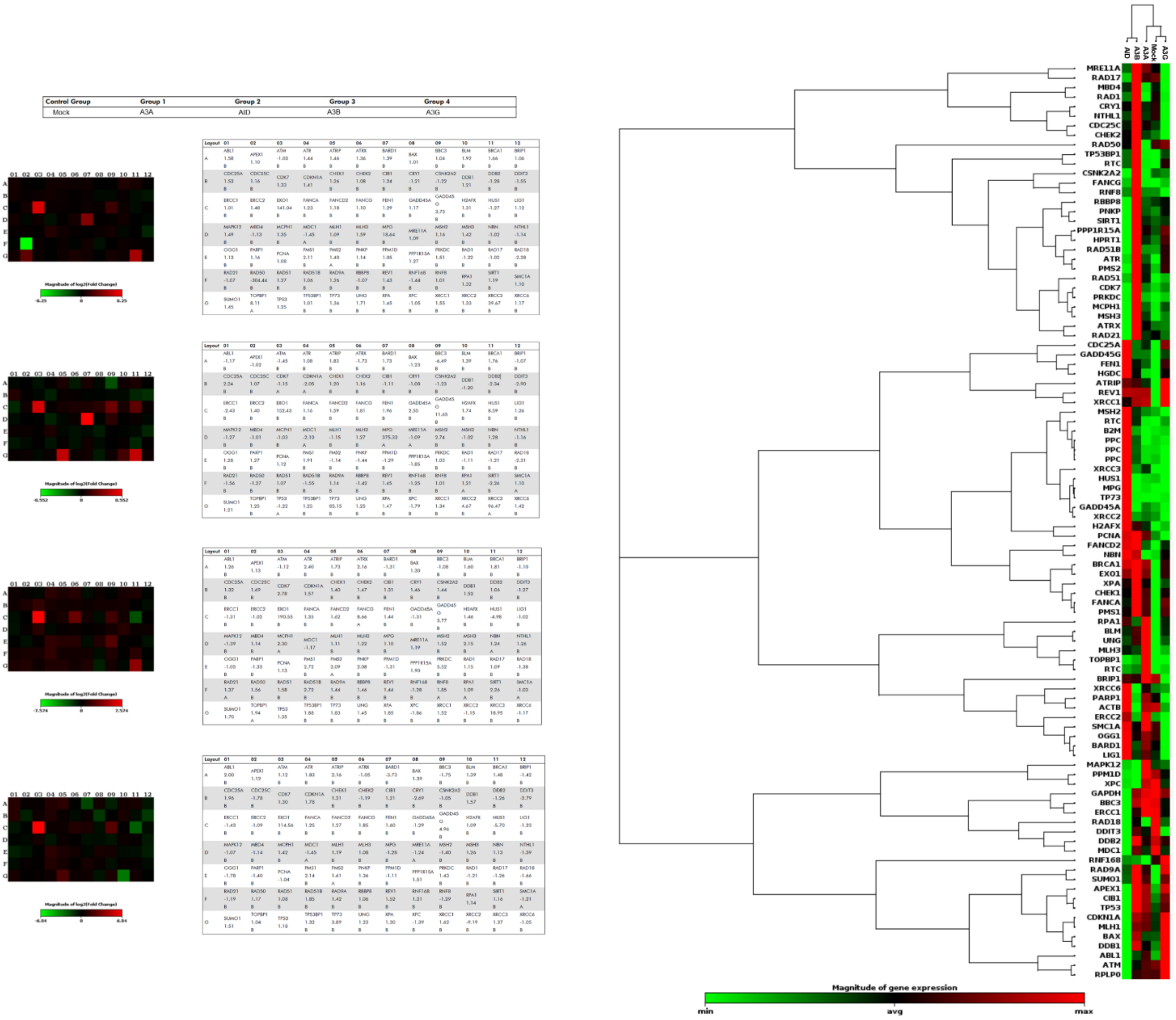

Figure S5. Microarray analysis and clustergram of HepG2-1.1merHBV cells transfected with CRISPRa targeting APOBEC/AID. Cells were transfected with dCas9-p300 targeting A3A, A3B, AID, A3G or with a non-targeting sgRNA (Mock control), and used for microarray profiling 3 days post transfection. Co-regulated genes across groups or individual samples are displayed at a heatmap with dendrograms (right side).

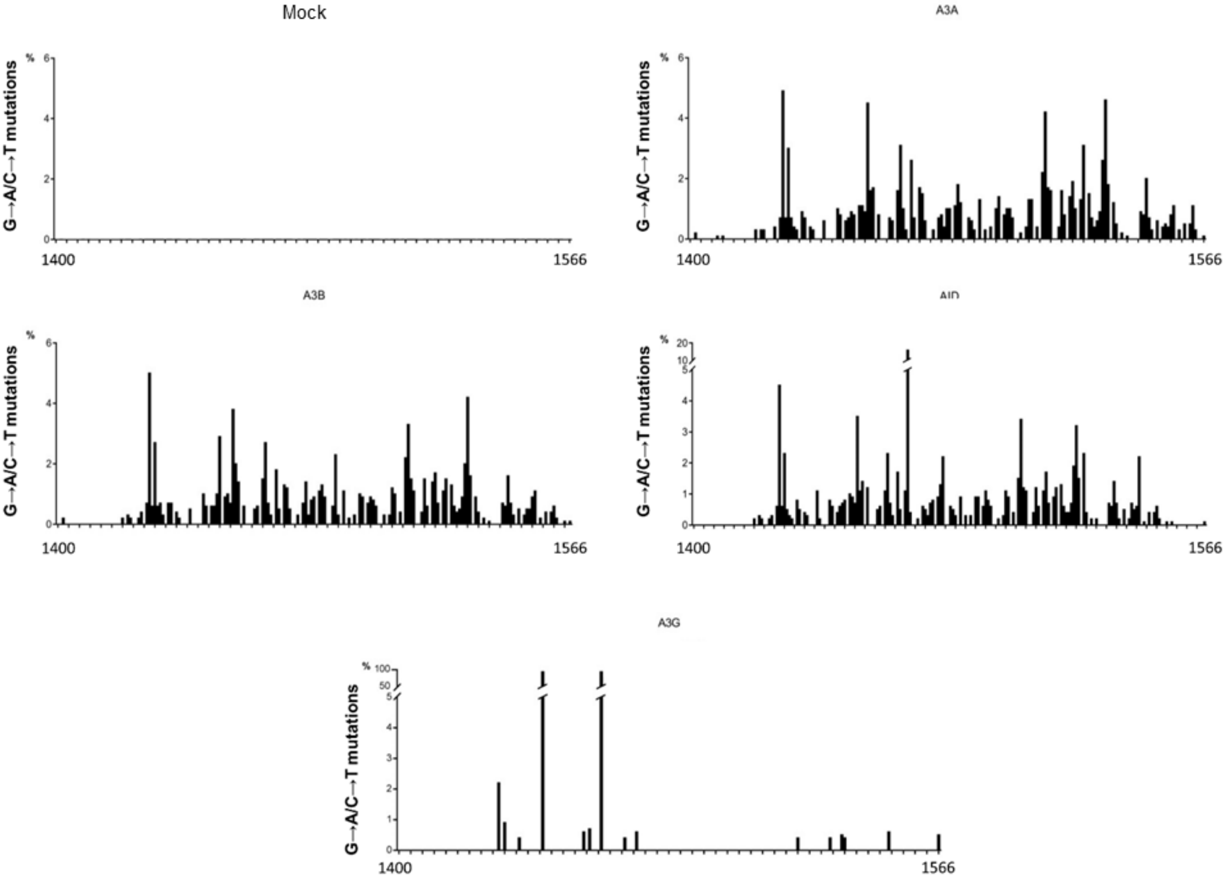

Figure S6. Deamination profile of HBV cccDNA upon APOBEC/AID activation by CRISPRa. HBx region of HBV genome (1400-1566 n.t.) was amplified with specified primers and analyzed by NGS. Deamination map indicates the frequency of G→A/C→T mutations at indicated positions. Mock, dCas9-p300 with a non-targeting sgRNA.

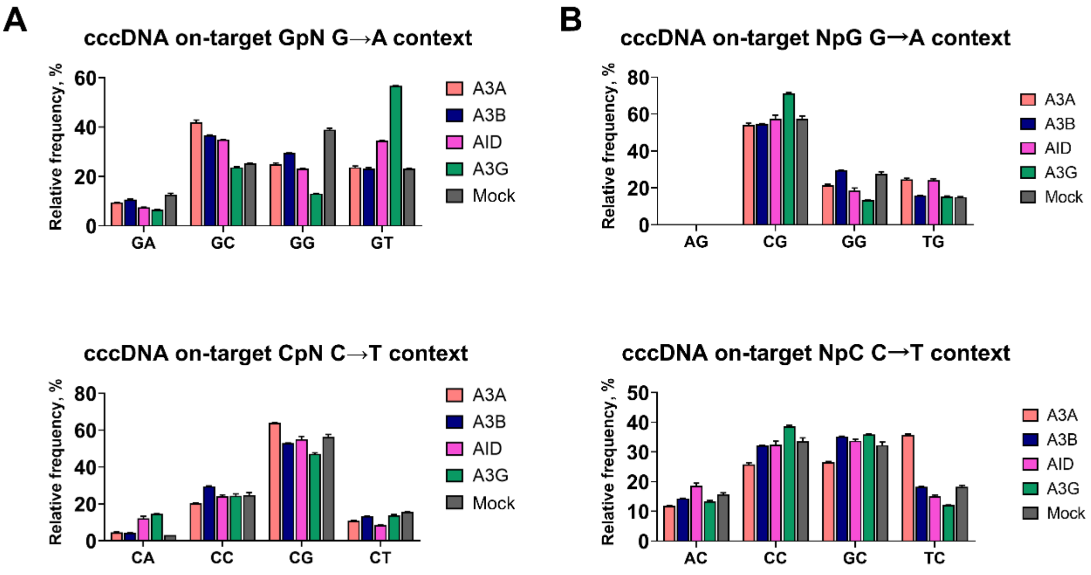

Figure S7. Analysis of cccDNA dinucleotide mutation context. (A) G/CpN context and (B) NpG/C context of G→A and C→T mutations upon CRISPR-activation of A3A, A3B, AID and A3G. Mock – dCas9-p300 system co-transfected with a non-targeting sgRNA. Analysis of NpC/G and C/GpN dinucleotide context showed that upon CRISPRa of A3A cccDNA harbored G→A substitutions mostly in 5’-GC-3’ and 5’-TG-3’ context (underlined base indicates a modified nucleotide), and C→T mutations in 5’-CG-3’ and 5’-TC-3’ context. A3B deaminated cccDNA preferentially in a 5’-GC-3’/5’-GG-3’ and 5’-CC-3’ sites. Frequent 5’-GC-3’/5’-GT-3’ and 5’-TC-3’/5’-AC-3’ mutations were apparent upon AID CRISPRa. A3G showed preference for 5’-GT-3’/5’-CG-3’ and 5’-CA-3’/5’-CC-3’ sites. Mock, dCas9-p300 with a non-targeting sgRNA.

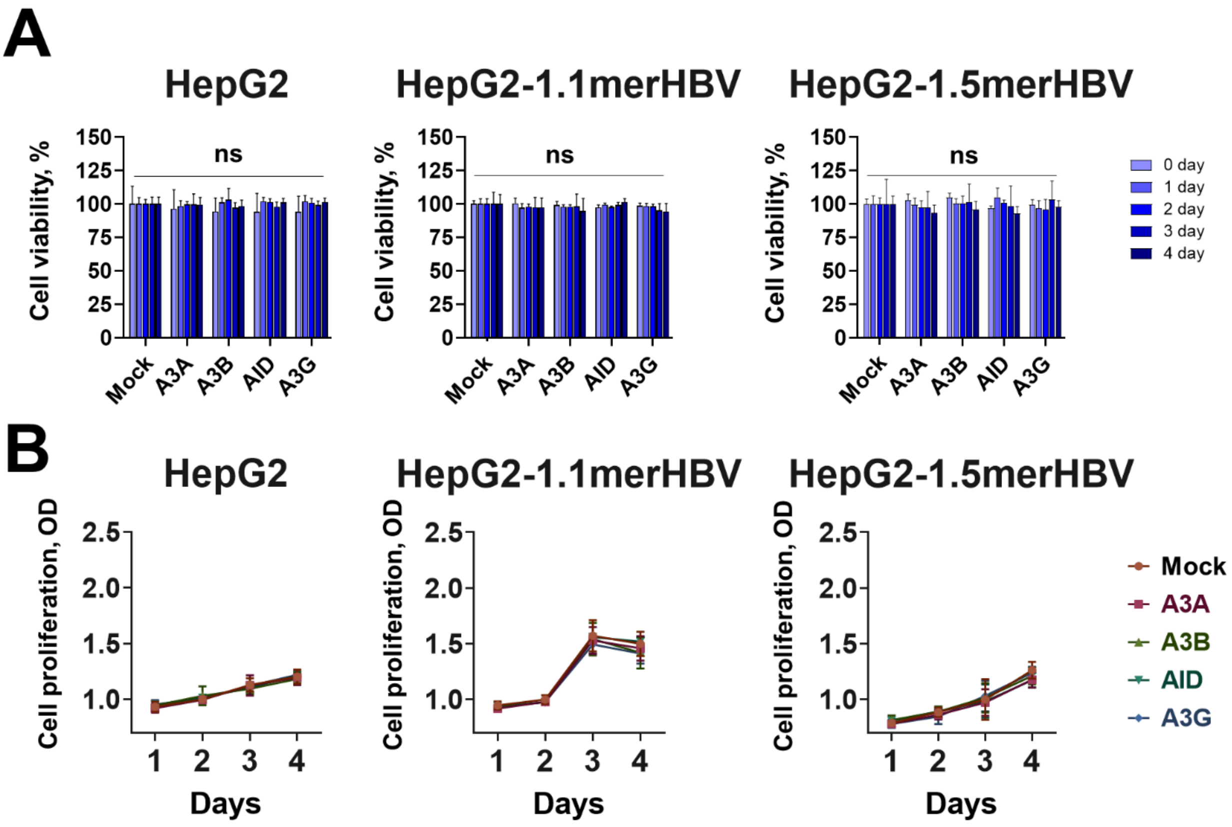

Figure S8. Analysis of APOBEC/AID toxicity. APOBEC/AID genes were activated in HepG2, HepG2-1.1merHBV or HepG2-1.5merHBV cell lines using CRISPRa. Cytotoxicity and proliferation of cells were measured at days 0 to 4 post transfection using Cytotoxicity assay kit (Abcam). Mock, dCas9-p300 with a non-targeting sgRNA. All changes are not statistically significant. *ns* – not significant.

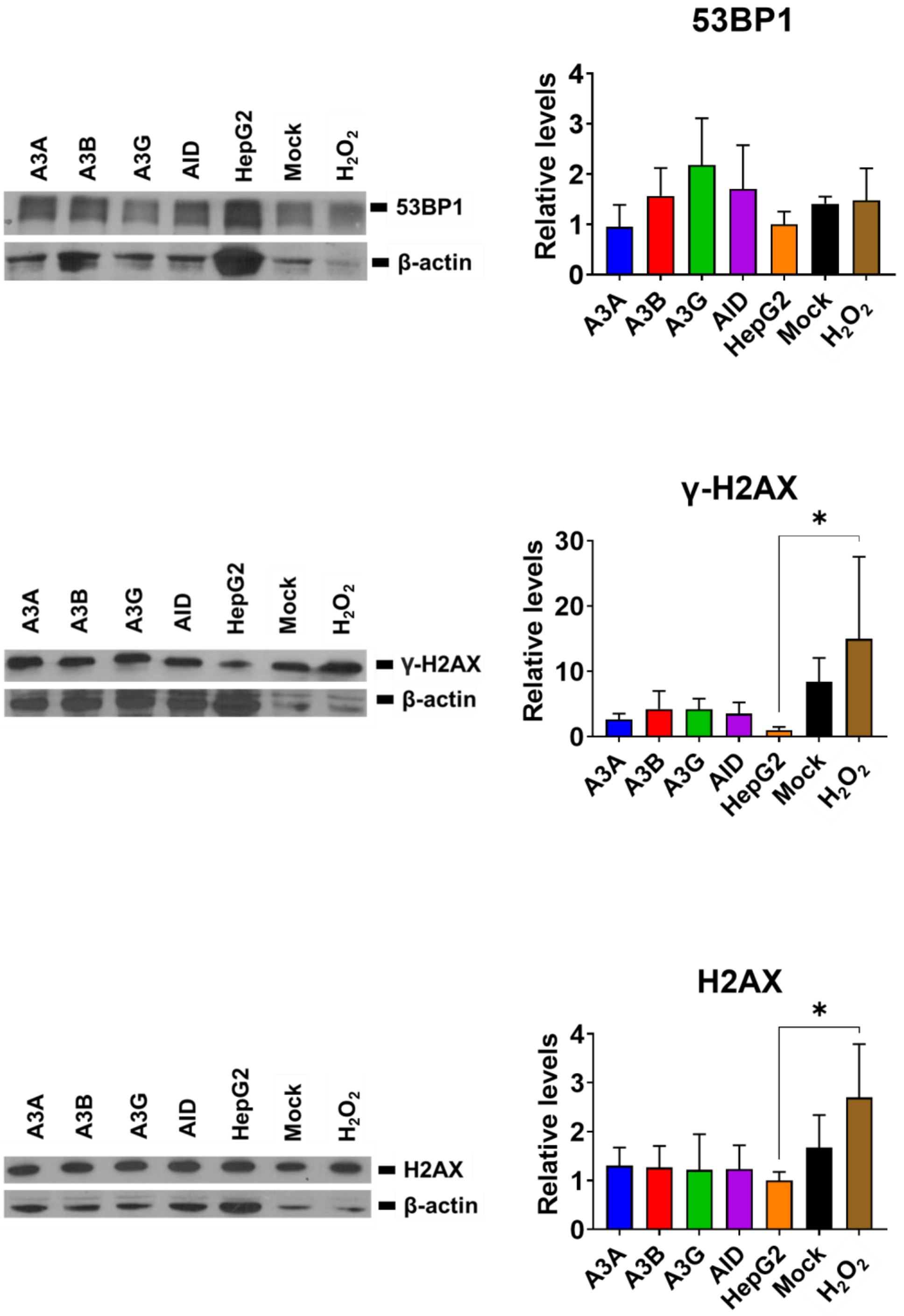

Figure S9. Western blot analysis of 53BP1, γ-H2AX, H2AX, and β-actin in CRISPRa-transfected cells with relative protein densities. Mock, dCas9-p300 with a non-targeting sgRNA.

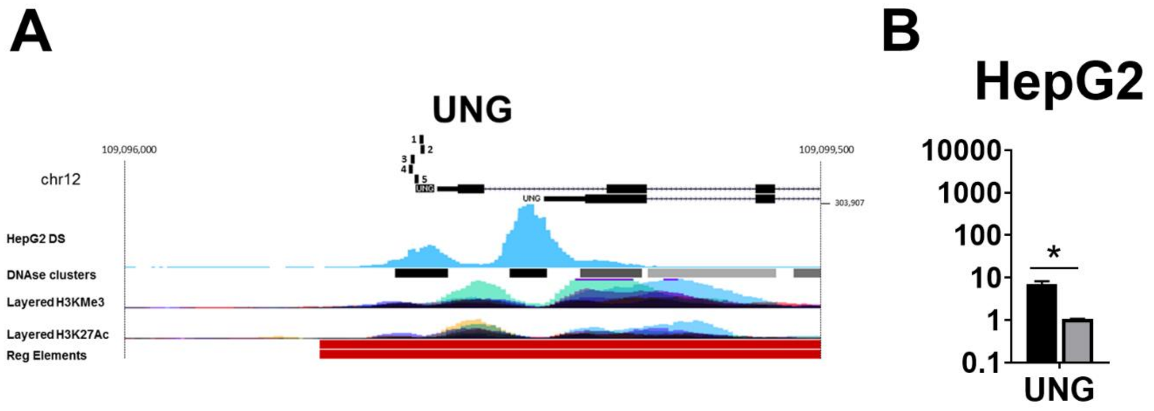

Figure S10. CRISPR-activation of UNG gene. (A) Design of sgRNA targeting UNG promoter region. (B) Peal activation of UNG using CRISPRa. UNG mRNA levels with targeting CRISPRa (black bars) or CRISPRa system with a mutant form of p300 (grey bars). UNG mRNA levels are relative to GAPDH mRNA. $^{\circ}p < 0.05$, $^{\Delta}p < 0.01$, $^{\#}p < 0.001$, $^{*}p < 0.0001$.

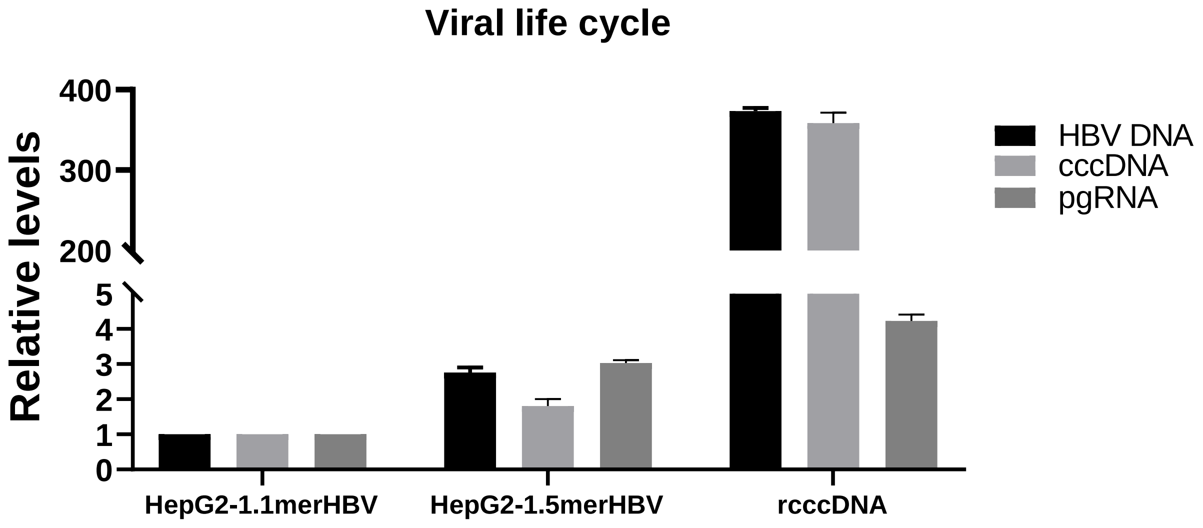

Figure S11. HBV replication at in vitro models. Relative HBV DNA, cccDNA and pgRNA levels were measured at 3 cell lines: HepG2-1.1merHBV with 24 hours of HBV tet-on promoter

activation using doxycycline, HepG2-1.5merHBV with constitutive production of HBV from a wild-type promoter and in HepG2 cells transfected with HBV rcccDNA.

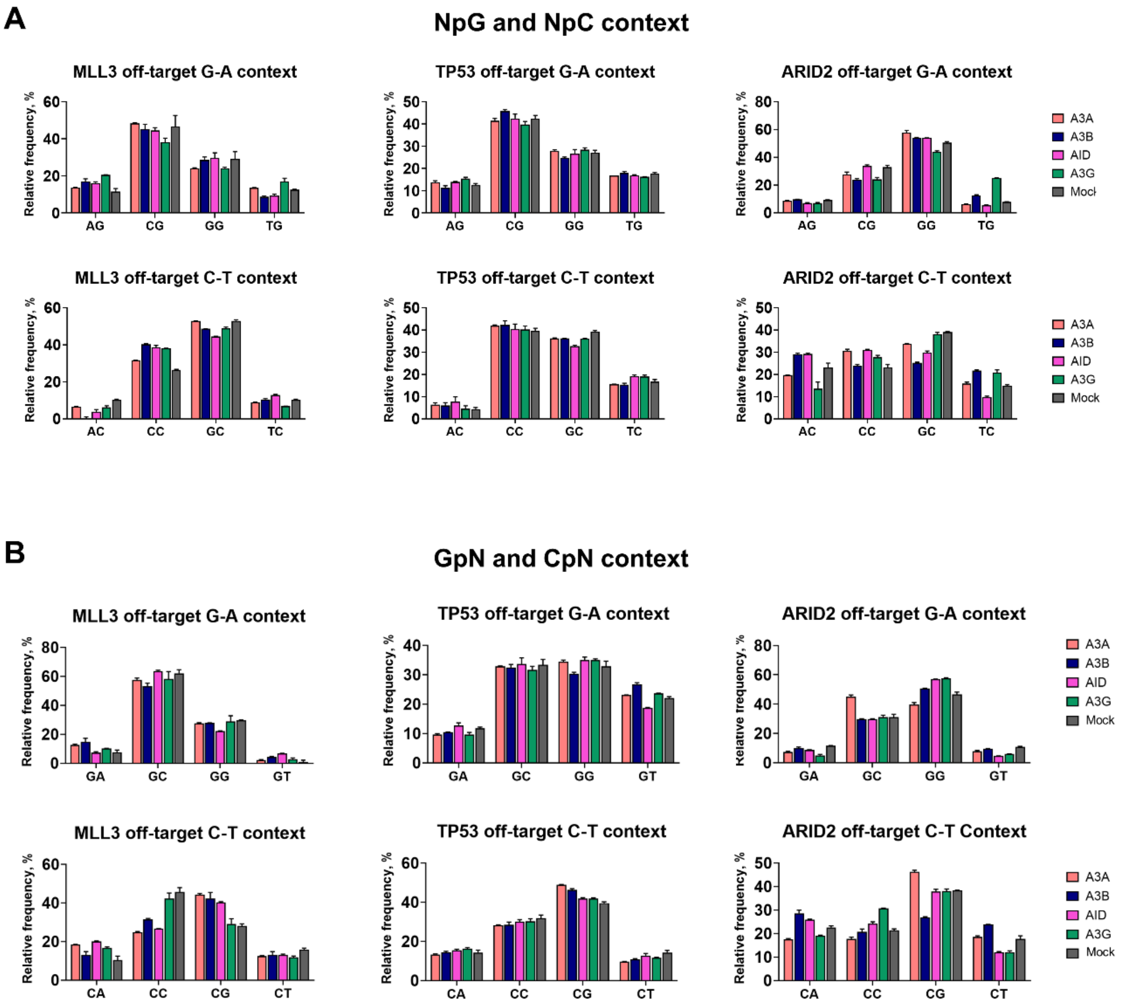

Figure S12. APOBEC/AID mutational context at genomic sites.

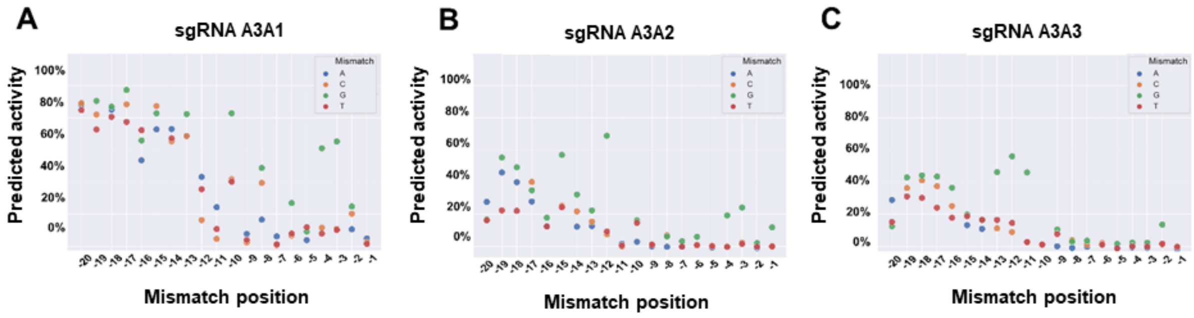

Figure S13. Predicted activity of attenuated sgRNAs for A3A gene.

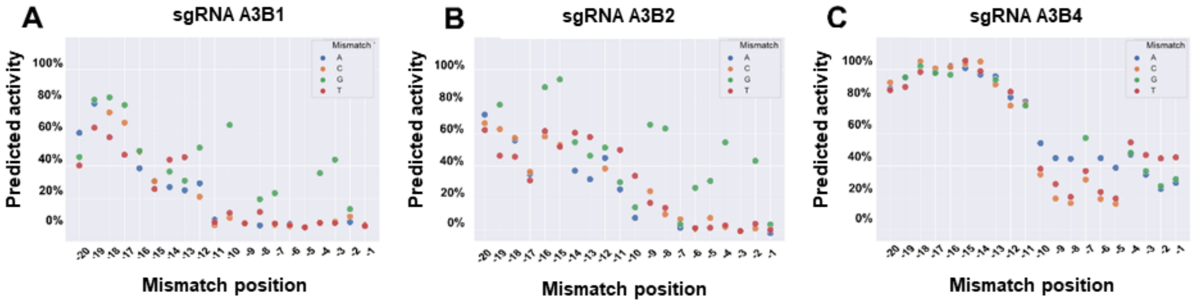

Figure S14. Predicted activity of attenuated sgRNAs for A3B gene.

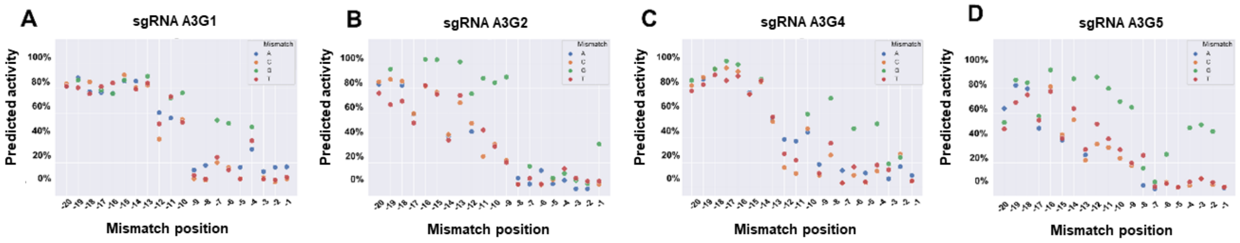

Figure S15. Predicted activity of attenuated sgRNAs for A3G gene.

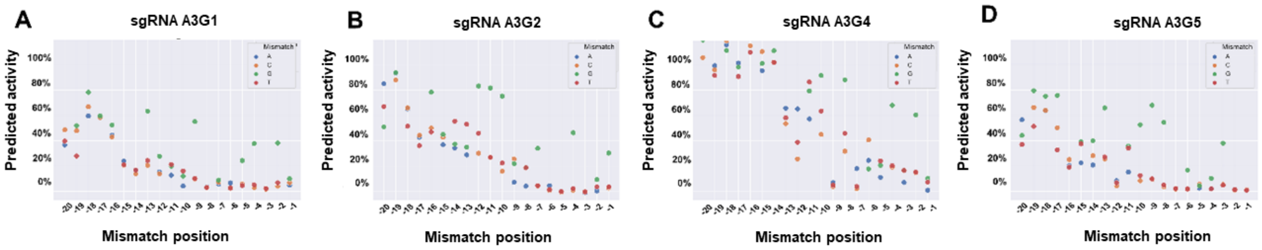

Figure S16. Predicted activity of attenuated sgRNAs for AID gene.

Tables S1-S17 and S20 are supplied as an excel file.

Table S18. Parameters of designed sgRNAs.

Name	5'→3' nucleotide sequence	Predicted efficacy	Chain	MM1	MM2	MM3	MM4	PAM
A3A1	GACACTGTGGAGTGACAATG	0,51	-	0	4	19	113	GGG
A3A2	CCCTCATTAGCGTCAGAACA	0,49	-	0	0	3	25	AGG
A3A3	CAAAACCAAAGCTTGCCCAG	0,7	+	0	2	19	93	AGG
A3A4	GCTAATGAGGGTGGCACACT	0,53	+	0	0	10	86	CGG
A3A5	GGCCCACAGGGAGCAAAGTG	0,65	+	0	2	21	129	TGG
A3B1	CCAATGCCTGAGCAGGAATG	0,81	+	1	5	18	118	GGG
A3B2	GAGGAACCTCCAATAAAGAC	0,55	-	1	0	11	51	AGG
A3B3	ATTGGAGGTTCCTCTGCCAG	0,85	+	0	1	14	77	CGG
A3B4	GCATTGGTGTGGGAGGCCCC	0,79	-	1	11	23	192	GGG
A3B5	CATTGCAGCCTGAGCCTGGG	0,71	-	0	2	33	210	AGG
AID1	ACCCCCACCAGGTACCCCAA	0,71	+	0	2	8	60	CGG
AID2	GATTTGGATCCAGTGAAGAT	0,72	+	0	1	9	71	GGG
AID3	CTGGATCCAAATCAGGAGCA	0,59	-	0	0	12	83	AGG
AID4	TGTCATAGGCAGAGTCACAC	0,66	+	0	0	6	91	AGG
AID5	CATAGCTAGCAAAGATCAGG	0,6	+	0	0	4	75	AGG
A3G1	CCCGCTTCCTGCTCAGGCGC	0,58	-	2	0	4	97	TGG
A3G2	GAGGAAGATAAAGCGTCCCA	0,66	+	0	0	7	32	GGG
A3G3	TAAAGACAGGCCGCTCTGTG	0,77	-	0	1	3	47	CGG
A3G4	AGAAAGAGGGTGAGAGACTG	0,62	+	0	14	116	401	AGG
A3G5	CCCCAGAGAAAACCAGAAAG	0,63	+	0	5	37	228	AGG

Table S19. Primers used for 3D-PCR analysis.

**Name**	**5'→3' nucleotide sequence**
3DcccDNA fw	GCCTATTGATTGGAAAGTATGT
3DcccDNA rev	AGCTGAGGCGGTATCTA
HBxin fw	ATGGCTGCTARGCTGTGCTGCCAA
HBxin rev	AAGTGCACACGGTYYGGCAGAT
