# Supplementary material for: Transient and tunable CRISPRa regulation of APOBEC/AID genes for targeting hepatitis B virus
Source: Mol Ther Nucleic Acids. 2023 Apr 20;32:478–93. doi: 10.1016/j.omtn.2023.04.016 (PMC10176074; doi:10.1016/j.omtn.2023.04.016)
Supplement: Document S2. Article plus supplemental information [file mmc3.pdf]

Molecular Therapy
Nucleic Acids
Original Article

Transient and tunable CRISPRa regulation of APOBEC/AID genes for targeting hepatitis B virus

Dmitry Kostyushev,[1,2,14] Sergey Brezgin,[1,2,14] Anastasiya Kostyusheva,[1,14] Natalia Ponomareva,[1,2,3] Ekaterina Bayurova,[4] Natalia Zakirova,[5] Alla Kondrashova,[4] Irina Goptar,[6] Anastasiya Nikiforova,[6] Anna Sudina,[7] Yurii Babin,[1,13] Ilya Gordeychuk,[4,8,9] Alexander Lukashev,[1] Andrey A. Zamyatnin, Jr.,[2,10,11,12] Alexander Ivanov,[5] and Vladimir Chulanov[2,8,9]

[1]Martsinovsky Institute of Medical Parasitology, Tropical and Vector-Borne Diseases, Sechenov University, 119991 Moscow, Russia; [2]Scientific Center for Genetics and Life Sciences, Division of Biotechnology, Sirius University of Science and Technology, 354340 Sochi, Russia; [3]Department of Pharmaceutical and Toxicological Chemistry, Sechenov First Moscow State Medical University, 119146 Moscow, Russia; [4]Chumakov Federal Scientific Center for Research and Development of Immune and Biological Products of Russian Academy of Sciences, 108819 Moscow, Russia; [5]Center for Precision Genome Editing and Genetic Technologies for Biomedicine, Engelhardt Institute of Molecular Biology, Russian Academy of Science, 119991 Moscow, Russia; [6]Izmerov Research Institute of Occupational Health, 105275 Moscow, Russia; [7]Federal State Budgetary Institution Centre for Strategic Planning and Management of Biomedical Health Risks of the Federal Medical Biological Agency, 119435 Moscow, Russia; [8]Institute for Translational Medicine and Biotechnology, Sechenov First Moscow State Medical University, 127994 Moscow, Russia; [9]Department of Infectious Diseases, Sechenov First Moscow State Medical University, 119146 Moscow, Russia; [10]Institute of Molecular Medicine, Sechenov First Moscow State Medical University, 119991 Moscow, Russia; [11]Belozersky Institute of Physico-Chemical Biology, Lomonosov Moscow State University, 119992 Moscow, Russia; [12]Faculty of Health and Medical Sciences, University of Surrey, Guildford GU2 7X, UK

APOBEC/AID cytidine deaminases play an important role in innate immunity and antiviral defenses and were shown to suppress hepatitis B virus (HBV) replication by deaminating and destroying the major form of HBV genome, covalently closed circular DNA (cccDNA), without toxicity to the infected cells. However, developing anti-HBV therapeutics based on APOBEC/AID is complicated by the lack of tools for activating and controlling their expression. Here, we developed a CRISPR-activation-based approach (CRISPRa) to induce APOBEC/AID transient overexpression (>4–800,000-fold increase in mRNA levels). Using this new strategy, we were able to control APOBEC/AID expression and monitor their effects on HBV replication, mutation, and cellular toxicity. CRISPRa prominently reduced HBV replication (~90%–99% decline of viral intermediates), deaminated and destroyed cccDNA, but induced mutagenesis in cancer-related genes. By coupling CRISPRa with attenuated sgRNA technology, we demonstrate that APOBEC/AID activation can be precisely controlled, eliminating off-site mutagenesis in virus-containing cells while preserving prominent antiviral activity. This study untangles the differences in the effects of physiologically expressed APOBEC/AID on HBV replication and cellular genome, provides insights into the molecular mechanisms of HBV cccDNA mutagenesis, repair, and degradation, and, finally, presents a strategy for a tunable control of APOBEC/AID expression and for suppressing HBV replication without toxicity.

INTRODUCTION

Chronic hepatitis B (CHB) is a global health threat, with over 250 million people chronically infected and ~1 million annual deaths from CHB outcomes (hepatocellular carcinoma and liver cirrhosis).[1] CHB is caused by infection of hepatocytes by hepatitis B virus (HBV), a small DNA virus with a complicated life cycle.[2] HBV is a virus from the Hepadnaviridae family, and has a partially double-stranded DNA genome (relaxed circular DNA [rcDNA]) with four overlapping open reading frames encoding viral proteins HBx, HBs (small, medium, and large surface proteins), HBc, HBe, and viral polymerase.[3] After infecting the cells, HBV virions are uncoated and transported into the nucleus where rcDNA is converted into double-stranded, covalently closed circular DNA (cccDNA). HBV cccDNA resides in nuclei of infected cells and serves as the template for all viral transcripts, including the pregenomic RNA (pgRNA).[4] HBV pgRNA is packaged into viral capsids in the cytoplasm and reverse-transcribed to rcDNA. Stability of HBV cccDNA ensures chronicity of infection; its highest persistence is supported by *de novo* infection[5,6] and intracellular

Received 25 October 2022; accepted 17 April 2023;
https://doi.org/10.1016/j.omtn.2023.04.016.

[13]Present address: MeLSyTech, Ltd, 603901 Dzerzhinsk, Nizhny Novgorod region, Russia

[14]These authors contributed equally

Correspondence: Dmitry Kostyushev, Laboratory of Genetic Technologies and Drug Development, Sechenov University, 119991 Moscow, Russia.
E-mail: dkostushev@gmail.com

 Crown Copyright © 2023
This is an open access article under the CC BY license (http://creativecommons.org/licenses/by/4.0/).

recycling of rcDNA.[7,8] Modern antivirals suppress viral replication, improving clinical outcomes, but do not inactivate HBV cccDNA.[9] Destroying HBV cccDNA may lead to sustained virological response and, potentially, CHB cure.[10]

A common mechanism of host defense against viral infections is activation of intracellular immune responses, which may include thousands of genes.[11] An important barrier to viral replication in human cells is a family of proteins that belong to a family of apolipoprotein B mRNA editing enzyme, catalytic polypeptide-like/activation-induced cytidine deaminases (APOBEC/AID).[12,13] These factors deaminate cytosine residues of nucleic acids, resulting in C→T and G→A mutations and, in some cases, degradation of viral genomes.[14] Many viruses developed elaborate mechanisms to inhibit APOBEC/AID deaminases.[15] A plethora of studies[16] showed that overexpression of APOBEC/AID suppresses HBV replication by editing HBV rcDNA, inhibiting viral transcription, packaging of viral pgRNA into capsids and reverse transcription, and even destroying HBV cccDNA.[17] APOBEC3A (A3A), APOBEC3B (A3B), and AID garnered particular attention, as they can be recruited to HBV cccDNA with the assistance of HBV's core protein (HBcAg), deaminating and destroying HBV cccDNA.[18] APOBEC3G (A3G) is regarded as the most potent HBV mutator, acting during reverse transcription of HBV,[19] but does not interact with HBV cccDNA. Crucially, A3A, A3B, and AID displayed no cellular toxicity, as HBcAg appears to divert them from interacting with host DNA.[18,20] This is particularly important because long-term overexpression of APOBEC/AID is notoriously toxic to cells,[21] resulting in frequent deamination of the host genome and chromosomal aberrations. Indeed, APOBEC/AID have long been recognized as important drivers of human cancers.[22]

Thus, a major challenge in developing a cure against CHB is safe activation of *APOBEC/AID*. Uncontrolled overexpression of *APOBEC/AID* from coding vectors inevitably damages DNA. Hence, tunable activation of *APOBEC/AID* genes would represent an optimal strategy for deactivating HBV cccDNA and clearing HBV from infected cells.

To directly activate genes of interest, a set of precise tools has been designed based on modified CRISPR-Cas9 systems, collectively termed dCas9 activation tools (CRISPRa).[23–26] In brief, CRISPRa consists of a dCas9 protein fused to a transcriptional activator, like the catalytic subunit of p300 acetyltransferase (a universal regulator of gene transcription).[27] A short single guide RNA (sgRNA) can target dCas9-p300 to host gene regulatory elements, activating transcription. Among the many advantages of CRISPRa, a major one is that the induced overexpression of target genes is more physiologic, as it is driven by endogenous regulatory elements.[23] Previously, CRISPRa-based approaches have been shown to be safe and effective for correcting genetic defects,[28] suppressing HIV[29] infection, etc.

Here, we designed CRISPRa systems for inducing expression of all major APOBEC/AID factors for targeting the HBV genome, and explored antiviral and potential toxic effects of CRISPRa-mediated *APOBEC/AID* gene activation. Overexpression of *APOBEC/AID* genes remarkably (>98%) reduced HBV replication, but at the same time induced off-site deamination of the host genes associated with cancer. Notably, deamination of the host genome occurred only in cells with low HBV replication levels, whereas upon high viral loads, host mutagenesis was not observed. To address the challenge of APOBEC/AID mutagenic activity, we took advantage of systematically attenuated sgRNAs (att-sgRNAs, harboring mismatches at specific positions) for controlling CRISPR-activation levels of target genes.[30] This enabled retention of robust antiviral activity and elimination of off-site mutagenesis and toxicity of CRISPRa-induced genes.

Here, we present a novel, effective anti-HBV approach based on *APOBEC/AID* controlled expression using CRISPRa ribonucleoproteins (RNPs) with att-sgRNAs that, for the first time, were employed to eliminate off-site genomic DNA modifications, paving the way for clinical development of innate immunity activation strategies. Our study also provides the first comprehensive evaluation of the effects of physiologically overexpressed *APOBEC/AID* on HBV replication and cellular and genomic toxicity.

RESULTS

Transient upregulation of target *APOBEC/AID* genes by CRISPRa

To upregulate expression of *APOBEC/AID* genes, we designed sets of five sgRNAs targeting promoters of each gene (Figure 1A) and identified sgRNAs that mediated the highest anti-HBV activity using an HBV recombinant cccDNA (rcccDNA) transfection model in HepG2 cells (Figure S1). HBV rcccDNA is commonly used to study HBV replication and test antiviral approaches.[31,32] *Streptococcus pyogenes* dCas9-p300-expressing plasmid (denoted as CRISPRa) was used for gene activation, a mutant version of dCas9-p300 plasmid with an inactivated p300 acetyltransferase and corresponding sgRNAs were used as control. CRISPRa with the most efficient sgRNAs suppressed HBV transcription by > 80%–90% (Figure S1). Using selected sgRNAs, we demonstrated robust (∼4–8 to >800,000-fold) activation of target gene transcription in both uninfected HepG2 cells, and HepG2-1.1merHBV and HepG2-1.5mer HBV cells with active viral replication (Figure 1B). We also observed enrichment of *APOBEC/AID* promoter regions with H3K27Ac, an epigenetic mark of transcriptionally active chromatin acetylated by p300, indicative of efficient acetylation of target gene promoters (Figure 1C).

Notably, transient transfection of CRISPRa resulted in activation of target gene transcription as early as 27–32 h post transfection (p.t.) and returning to baseline levels by 48 h p.t. (Figure 1D). In general, elevated levels of target gene mRNAs were observed for as long as ∼12–20 h. Epigenetic marks modified by p300 are unstable and are rapidly reversed due to epigenetic memory effect, returning gene transcription to baseline levels.[33] Inducing *APOBEC/AID* mRNA levels was followed by increased levels of translated proteins by 2–3 days p.t. that waned at later time points (Figures 1E, S2, and S3).

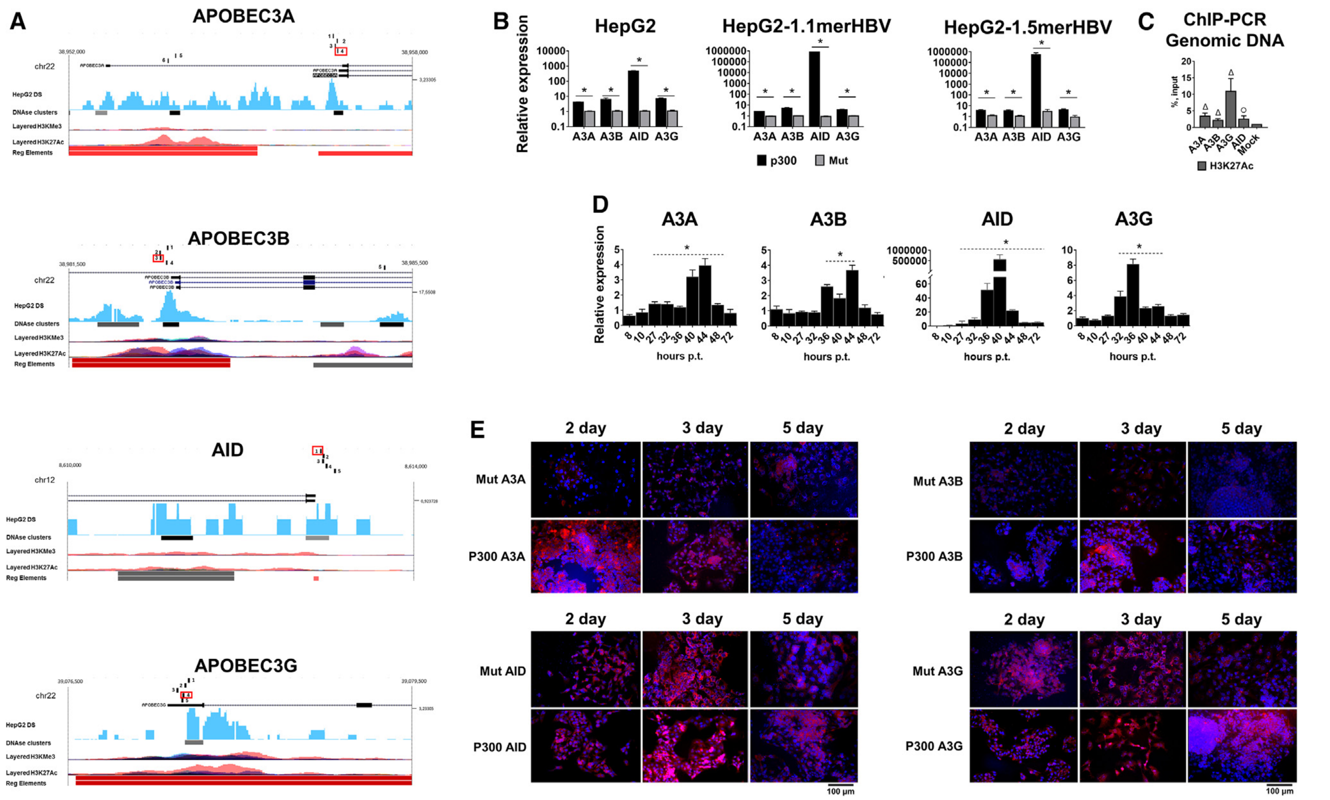

Figure 1. Activation of APOBEC/AID gene expression using CRISPRa

(A) Design of targeting sgRNAs. Digits 1 through 5 indicate locations of sgRNAs targeting a gene of interest (UCSC genome browser data). sgRNAs used in further studies are marked with red. (B) Peak mRNA levels of *APOBEC/AID* in cell lines transfected with targeting CRISPRa (black bars) or CRISPRa with a mutant form of p300 (gray bars). (C) Enrichment of *APOBEC/AID* promoter regions with H3K27Ac upon CRISPRa of target genes. (D) Dynamic analysis of *APOBEC/AID* mRNA levels relative to GAPDH mRNA after transfection of CRISPRa systems in HepG2-1.5merHBV cells (plotted relative to mock-treated control). Cells were transfected with CRISPRa plasmids and harvested at indicated timepoints post transfection. (E) Dynamic analysis of APOBEC/AID expression by immunocytochemistry in HepG2 cells. Mut, a mutant form of dCas9-p300 with sgRNA; p300, dCas9-p300 with sgRNA. Cells were stained for the corresponding APOBEC/AID proteins on specified days; cell nuclei are labeled with Hoechst33342 (blue). The results represent the mean of at least three experiments ± SD. $^{\circ}p < 0.05$; $^{\Delta}p < 0.01$; $^{\#}p < 0.001$; $^{*}p < 0.0001$.

Upon activation, APOBEC/AID displayed cell-wide, cytoplasmic, and nuclear distribution, typical for APOBEC/AID based on published studies,[34–36] and increased target protein expression in groups with transcriptionally activated genes (Figure S3). Hence, we demonstrated that CRISPRa can effectively induce transcription of *APOBEC/AID* genes in HBV-positive and -negative cell lines, resulting in transient elevation of mRNA and protein levels.

Strong suppression of HBV replication by APOBEC/AID

APOBEC/AID genes used in this study have a well characterized anti-HBV activity. CRISPR activation of each *APOBEC/AID* gene reduced HBV pregenomic RNA (pgRNA), the major transcript of the virus, and S-mRNA levels (mRNA molecules encoding HBV surface antigens) by ∼50%–80% (Figures 2A and 2B) and HBV DNA/cccDNA levels by ∼20%–70% (Figures 2C and 2D), HBsAg was slightly reduced only upon *A3A* activation (Figure 2E). While A3A, A3B, and AID can directly interact with and deaminate HBV cccDNA, A3G does not directly impact HBV cccDNA.[37] Indeed, CRISPRa of *A3A*, *A3B*, and *AID* genes resulted in substantial decline in HBV cccDNA levels, whereas *A3G* did not affect HBV cccDNA levels (Figure 2D).

To verify this effect, we activated *APOBEC/AID* genes in the HepG2-1.1merHBV cell line, in which HBV cccDNA is produced *de novo*, and in the HepG2-1.5merHBV cell line, in which HBV is constitutively expressed. In HepG2-1.1merHBV cells, *A3G* overexpression reduced HBV cccDNA *de novo* formation, whereas in HepG2-1.5merHBV cells with stable HBV expression, A3G did not affect pre-established HBV cccDNA levels (Figure S4).

Quantitative analysis of HBcAg-positive cells indicated complete elimination of HBcAg expression upon CRISPRa, whereas ∼10% of HBcAg-positive cells were detected in mock control groups (Figures 2F and 2G). Altogether, these results indicate that even very transient activation of *APOBEC/AID* potently inhibits HBV transcription and replication and (except for A3G) reduces HBV cccDNA.

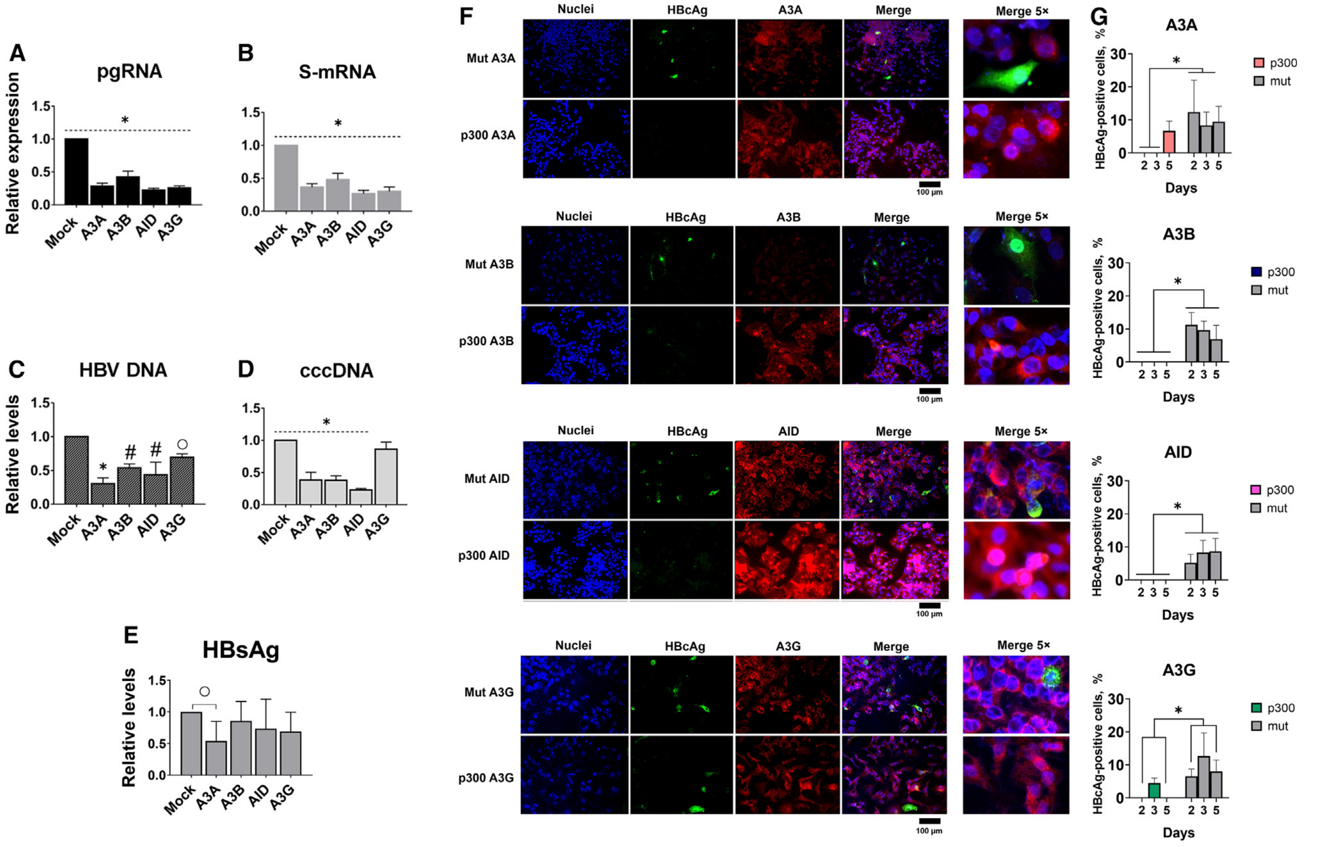

Figure 2. Anti-HBV activity of *APOBEC/AID* induced by CRISPRa

Suppression of HBV (A) pgRNA, (B) S-mRNA, (C) total intracellular HBV DNA, (D) cccDNA, and (E) HBsAg levels by CRISPRa in HepG2 cells co-transfected with rcccDNA. (F) Reduction in HBcAg expression by CRISPRa. HepG2 cells were co-stained for HBcAg (green) and corresponding APOBEC/AID protein (red). Cell nuclei were counterstained with Hoechst33342 dye. (G) Semi-quantitative analysis of HBcAg expression in experimental groups. HBV DNA and cccDNA levels are relative to β-globin levels; pgRNA and S-mRNA levels are relative to GAPDH mRNA levels. Mock, dCas9-p300 with a non-targeting sgRNA. The results represent the mean of at least three experiments ±SD. $^{\circ}p < 0.05$; $^{\Delta}p < 0.01$; $^{\#}p < 0.001$; $^{*}p < 0.0001$.

Deamination of HBV genomes by a CRISPRa approach

APOBEC/AID proteins can deaminate single-stranded regions of HBV DNA and cccDNA, leading to G→A and C→T mutations, while A3B was also shown to deaminate transcriptionally silenced, double-stranded cccDNA. Heavily edited viral genomes can be destroyed, likely with the assistance of uracil DNA glycosylase (UNG) and interferon-stimulated gene 20 (ISG20) proteins.[18,38] Otherwise, deleterious mutations may functionally inactivate HBV genomes. Deamination can be effectively detected by a 3D-PCR method, a common method used for assessing deamination in DNA,[18] which selectively amplifies A:T mutations in a C:G-rich HBx region of the HBV genome. The lower the temperature where the target amplicon can be produced, the higher is the deamination of the target.

We performed 3D-PCR analysis of HBV cccDNA isolated 2–5 days p.t., and observed additional PCR products of the expected size in CRISPRa-treated cells (Figure 3A). HBV cccDNA was purified from other genomic forms using a highly efficient T5 exonuclease procedure.[7,39] 3D-PCR results indicated extensive deamination of the target HBV cccDNA region. However, deamination kinetics differed across APOBEC/AID samples: A3A and A3B prominently deaminated HBV cccDNA at day 4 p.t., while deamination induced by AID and A3G was most evident 3 days p.t. Deaminated HBV cccDNA templates were virtually absent 5 days p.t. Semi-quantitative 3D-PCR produced similar results (Figure 3A). These data indicate different kinetics in the deamination of HBV cccDNA by APOBEC/AID, and suggest that heavily deaminated viral genomes are destroyed.

To directly assess degradation of HBV cccDNA by APOBEC/AID, we co-transfected uracil glycosylase inhibitor (UGI; uracil glycosylase is responsible for degradation of deaminated cccDNA) and measured HBV cccDNA levels and deamination. As expected, co-transfection of UGI blocked HBV cccDNA degradation and increased formation of 3D-PCR products at lower temperatures, indicative of heavy deamination of HBV cccDNA (Figure 3B).

Transcriptomic profiling of APOBEC/AID experimental groups in HepG2-1.1merHBV cells (this cell line was chosen because of

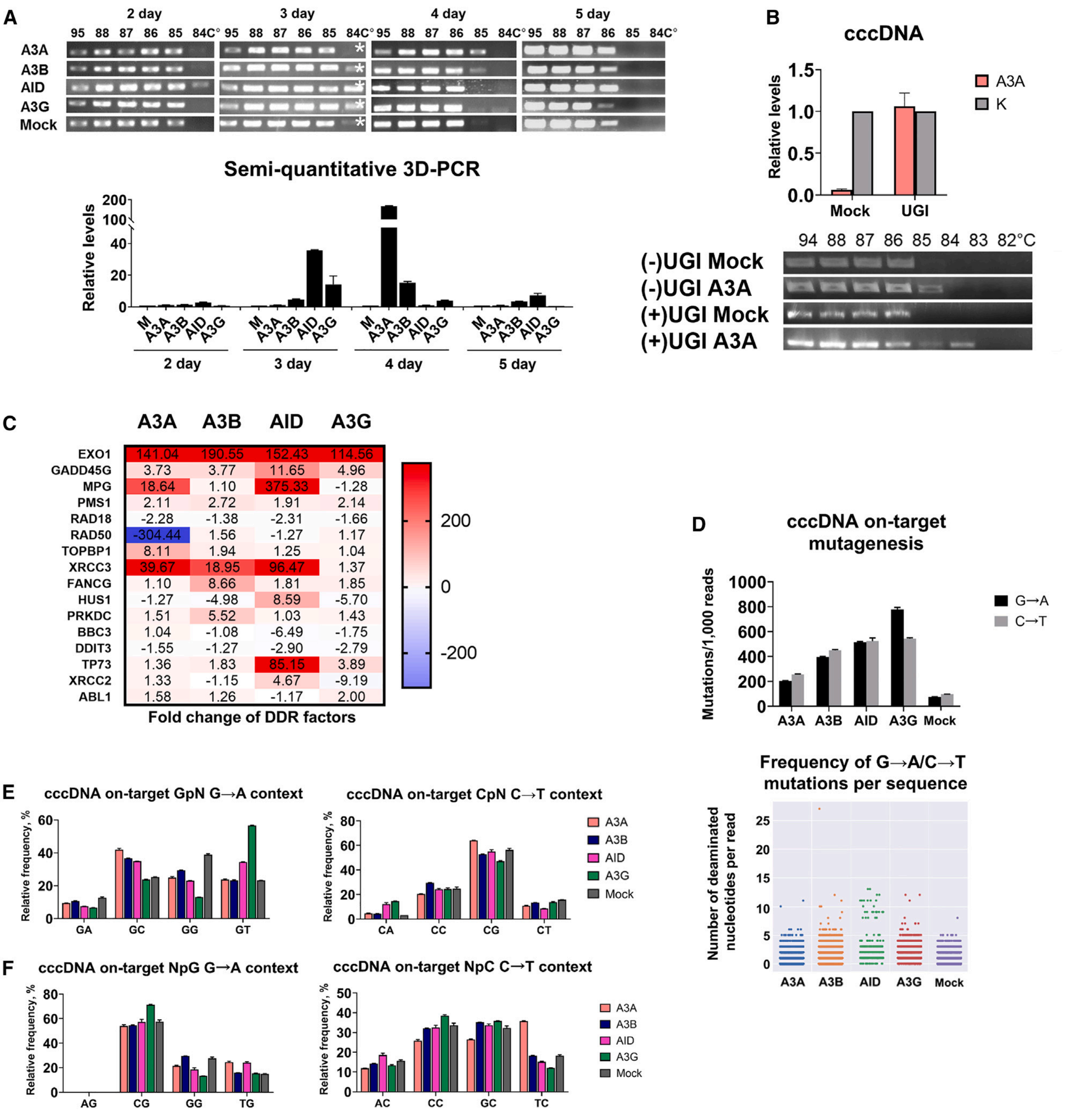

Figure 3. CRISPR activation of *APOBEC/AID* induces deamination of HBV cccDNA

(A) Analysis of on-target HBV cccDNA deamination by 3D-PCR in HepG2 cells transfected with rcccDNA (top). HBV rcccDNA was isolated at days 2–5 post transfection and analyzed by 3D-PCR. Generation of an additional amplicon at lower melting temperatures in the experimental conditions compared with control indicates deamination of the target site in cccDNA. Asterisks indicate PCR products selected for NGS analysis. Semi-quantitative 3D-PCR analysis of HBV cccDNA calculated by ddCt method where dCt is $2^{(Ct95^{\circ}C - Ctlow^{\circ}C)}$, and *low* is the lowest denaturation temperature (bottom), relative to mock control. Values are expressed as the mean of three independent experiments ±SD. (B) Inhibition of HBV cccDNA degradation by UGI: relative cccDNA levels (top) and 3D-PCR electrophoresis (bottom) results. HepG2 cells were co-transfected with rcccDNA, CRISPRa, and UGI-encoding vector or empty vector (no UGI); 3D-PCR was performed at day 4 post transfection. (C) Key transcriptomic changes defined by microarray profiling of HepG2-1.1merHBV cells with CRISPRa in HepG2 cells at day 3 post transfection. Color intensity is defined by the mean relative expression fold change. Complete heatmap is provided in Figure S4. (D) Frequency of (left) total G→A and C→T mutations and (right) G→A/C→T mutations per sequence in HBV cccDNA. HBV cccDNA was analyzed at day 4 post transfection. Mutation context of G→A/C→T mutations in HBV cccDNA in a (E) G/CpN and (F) NpG/C dinucleotide context. PCR results represent the mean of at least three experiments ±SD. Mock, dCas9-p300 plasmid with a non-targeting sgRNA.

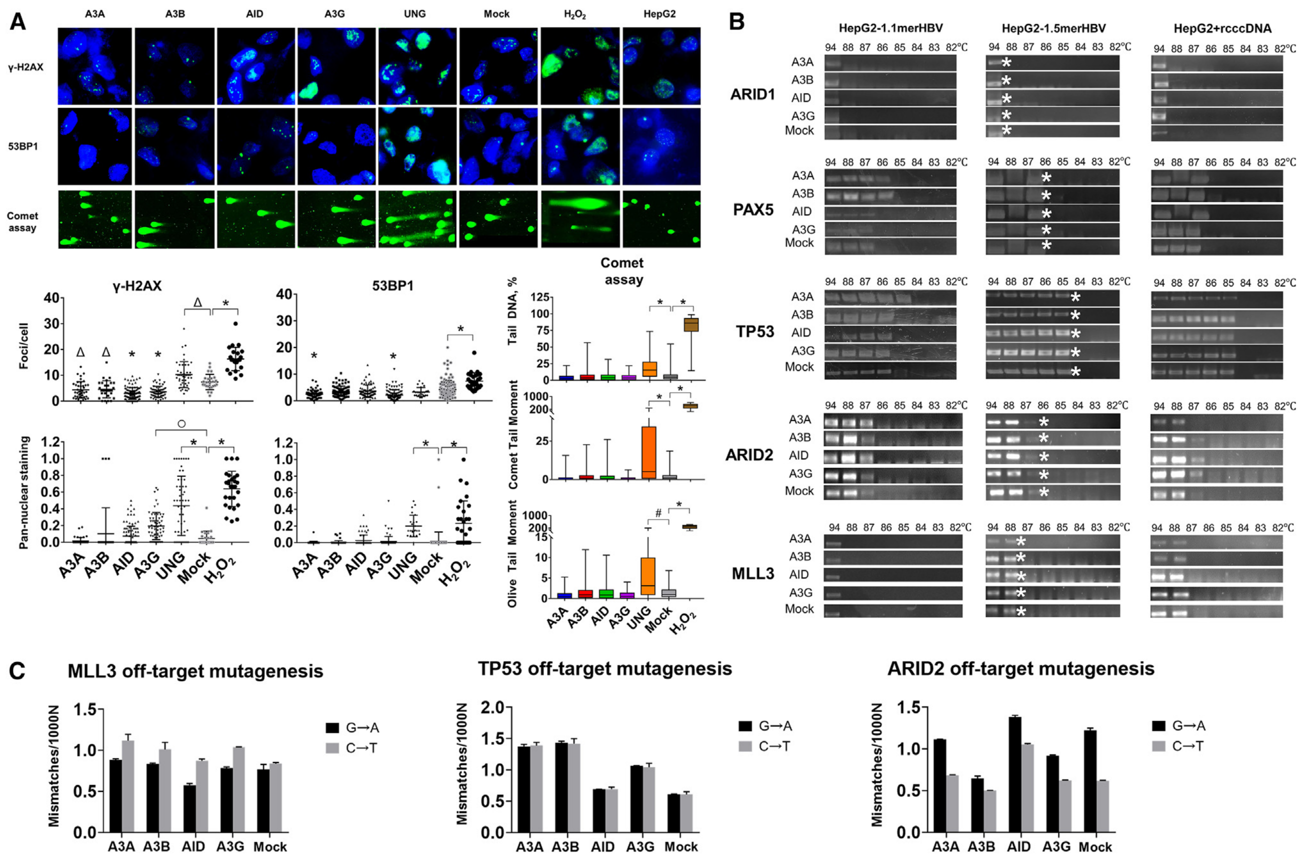

Figure 4. Toxicity and off-target effects of APOBEC/AID upon transient CRISPR activation

(A) Genotoxicity measured by immunostaining for γ-H2AX and 53BP1 and by comet assays; representative fluorescent images (top) and semi-quantitative analysis (bottom). HepG2 cells were stained for γ-H2AX or 53BP1 protein (green); cell nuclei were labeled with Hoechst33342 (blue). Each dot on the graphs represents the mean number of γ-H2AX/53BP1 foci per cell or pan-nuclear cells in the visible area. (B) 3D-PCR analysis of off-target deamination in the host genome. (C) NGS analysis of HepG2-1.5merHBV genomic regions. Mock, dCas9-p300 with a non-targeting sgRNA, used as control; H_2O_2, cells treated with hydrogen peroxide solution, used as a positive genotoxic control; untreated HepG2 cells were used as a negative control. All experiments were performed in triplicate, the results represent mean ± SD. $^{\circ}p < 0.05$; $^{\Delta}p < 0.01$; $^{\#}p < 0.001$; $^{*}p < 0.0001$.

more physiological levels of cccDNA during 24-h induction of tet-on promoter with doxycycline) at day 3 p.t. showed significant differences in the expression of DNA damage response factors (Figures 3C and S5). The most upregulated genes observed across experimental groups included *EXO1* (>100-fold increase), *XRCC3* (>19-fold increase in A3A, A3B, and AID groups), and *MPG* (∼19-fold increase in A3A and ∼350-fold increase in AID groups), which are responsible for DNA mismatch repair, homologous recombination, and base-excision repair, respectively. AID activation also significantly increased mRNA levels of factors involved in apoptosis, namely *GADD45G* (∼12-fold increase) and *TP73* (∼85-fold increase). Analysis of potential off-targets for each sgRNA used demonstrated that these genes are not the targets for dCas9-p300 and thus cannot be directly affected by dCas9-p300 (Table S1). As such, the observed transcriptional changes are directly related to APOBEC/AID activity. Moreover, a previous study by Matharu et al. using genome-wide chromatin immunoprecipitation sequencing (ChIP-seq) showed that CRISPRa is highly specific and does not induce overt changes in non-target genes.[40]

Next, we sequenced 3D-PCR products for each sample on day 3 p.t. to analyze HBV cccDNA mutagenesis and directly compare deaminating efficiency of each APOBEC/AID upon CRISPRa. Deep sequencing revealed frequent G→A and C→T mutations located throughout the analyzed region, with similar hotspots in A3A and A3B groups and distinct hotspots in A3G and AID groups (Figures 3D and S6). Frequency of G→A and C→T mutations was similar in A3A, A3B, and AID groups, while G→A mutations predominated upon A3G overexpression. Analysis of dinucleotide mutations at C/GpN and NpC/G sites revealed a characteristic mutation pattern (Figures 3E, 3F, and S7). The unique pattern of A3G-mediated deamination (Figures 3D and S6) and its effects on HBV cccDNA (Figures 2C and 2D) might indicate that the observed mutations arise from HBV cccDNA formed *de novo* from a deaminated

HBV rcDNA precursor.[17] In general, APOBEC/AID induced by CRISPRa resulted in pronounced deamination of the HBV genome.

Safety of transient *APOBEC/AID* activation in HBV *in vitro* models

APOBEC/AID substantially contribute to mutations driving development of many types of cancer.[22] APOBEC/AID factors can directly induce DNA damage, including double-stranded DNA breaks (DSBs), the most pernicious type of DNA damage.[41] Thus, we extensively analyzed cell toxicity, DNA damage, and host mutagenesis upon transient CRISPRa of *APOBEC/AID* genes. Cell viability and proliferation were not affected by CRISPRa activation of *APOBEC/AID* (Figure S8). To analyze the effects of APOBEC/AID on cellular genome integrity, we utilized the comet assay, which measures DNA breaks in single cells embedded in agarose gel; the characteristics of comet tails indicate the extent of DNA damage. Using comet assays and immunostaining analysis of γ-H2AX (a marker of DNA breaks) and 53BP1 proteins (a factor of DSBs repair signaling), we showed that *A3A*, *A3B*, and *AID* did not affect genomic stability (Figures 4A and S9). Moreover, APOBEC/AID reduced the mean number of γ-H2AX foci per cell, while A3A and A3G also reduced the mean number of 53BP1 foci per cell. These effects have been related to suppression of HBV replication and decreased DNA-damaging activity of the virus.[42] In contrast, A3G generated a significantly higher amount of pan-nuclear γ-H2AX foci (marker of clustered DNA lesions[43]). On the other hand, CRISPRa of *UNG* (Figure S10), a factor involved in base-excision repair of deaminated nucleotides, as well as addition of hydrogen peroxide (H_2O_2), a genotoxic agent serving as a positive control, inflicted significant DNA damage compared with a mock control (Figure 4A). Initially, we expected to use *UNG* activation to accelerate degradation of deaminated HBV cccDNA, but the high genotoxicity of UNG in HepG2 cells prompted us to abandon this strategy.

Several studies previously demonstrated that A3A, A3B,[18] and AID[44] bind to HBV's HBc protein, which directly targets deaminases to HBV cccDNA and thus reduces the damage to the host genome, restricting toxicity and modifications of genomic DNA. These data suggested that some APOBEC/AID enzymes are potential strategies for curing HBV infection, able to destroy the HBV genomic reservoir without harming host cells. However, these studies evaluated off-target mutagenesis in DNA regions that interact with the HBc protein; studies of clinically relevant genes mutated in human cancers are limited.[45] Second, it is unclear if off-site mutagenesis occurs when viral loads are low (either due to antiviral treatment or low viral replication) and HBc cannot effectively shield the genome from deaminases. To address these questions, we assayed mutagenic activity of the CRISPRa approach using different viral loads in three cell lines: HepG2-1.1merHBV (low-level HBV replication), HepG2-1.5merHBV (nearly physiological replication), and HepG2 co-transfected with HBV rcccDNA (high replication) (Figure S11). We then analyzed the CpG-rich regions of genes implicated in APOBEC/AID-driven carcinogenesis: *ARID1*, *PAX5*, *TP53*, *ARID2*, and *MLL3*.[22]

We observed off-target deamination of the *PAX5* gene by A3A and A3B and of the *TP53* gene by A3A in HepG2-1.1merHBV cells (Figure 4B). Additional 3D-PCR product was also registered in the *PAX5* gene with CRISPRa of *A3B* and *A3G* in HepG2-1.5merHBV cells. Off-target deamination was not observed in rcccDNA-transfected HepG2 cells. These data suggest an inverse relationship between HBV replication rates and side effects of APOBEC/AID enzymes. To analyze host mutagenesis directly, we deep-sequenced amplicons with the same melting temperature (not heavily mutated PCR products) in HepG2-1.5merHBV cells, a more physiologic model of HBV. Although additional 3D-PCR products were observed in *PAX5* only when *A3B* was used, deep sequencing identified a substantial increase in characteristic G→A/C→T mutations of the *TP53* gene (*A3A*, *A3B*, and *A3G* activation) and *ARID2* gene (*AID* activation) (Figures 4C and S12).

Collectively, transient CRISPRa of *A3A*, *A3B*, and *AID* does not induce significant DNA breaks in the host genome, while *A3G* activation does, increasing formation of cells with pan-nuclear γ-H2AX staining. HBV replication does in fact restrain APOBEC/AID from deaminating the host genome, but this effect declines upon reducing HBV viral loads.

Tunable regulation of *APOBEC/AID* activation by att-sgRNAs

APOBEC/AID is one of the very few factors that can directly mutate and destroy HBV cccDNA. Nevertheless, altered and prolonged *APOBEC/AID* overexpression drives many human cancers.[46] To hone the CRISPRa approach and make it safer for use in HBV-infected cells, we installed a new layer of regulating *APOBEC/AID* by CRISPRa.

A machine learning approach previously showed that introducing single-nucleotide mismatches into the 20-nt region of sgRNA enables titration of CRISPRa activity from 90% to 0% in 10% increments.[30] We designed libraries of att-sgRNAs with mismatched nucleotides targeting *APOBEC/AID* genes (Figures 5A and S13–S16, and Tables S2–S16) and tested their effects on mRNA levels (Figure 5B) and antiviral properties of the system (Figure 5C). In general, titration of mRNA levels followed the predicted model, with decreases of 10%–20% and up to 50% achieved by using att-sgRNAs. Antiviral activity of A3B, A3G, and AID was mostly unaltered even upon substantial (>2- to 20-fold) decline in target gene activation, whereas anti-HBV activity of A3A was consistently reduced compared with perfectly matched sgRNAs (Figures 5B and 5C). CRISPRa of APOBEC/AID induced deamination of HBV cccDNA with the majority of att-sgRNAs (measured by 3D-PCR). Thus, even attenuated activation of *APOBEC/AID* induces commensurate antiviral activity and deamination of the HBV genome (Figure 5D).

To explore whether attenuated activation of *APOBEC/AID* can suppress HBV replication without side effects, we analyzed how reduced CRISPR-mediated activation correlates with AID-associated toxicity (measured by upregulation of TP73 mRNA levels). When *AID* was activated by CRISPRa with a set of att-sgRNAs, TP73 mRNA levels correlated with *AID* expression (r = 0.8391) (Figure 5E).

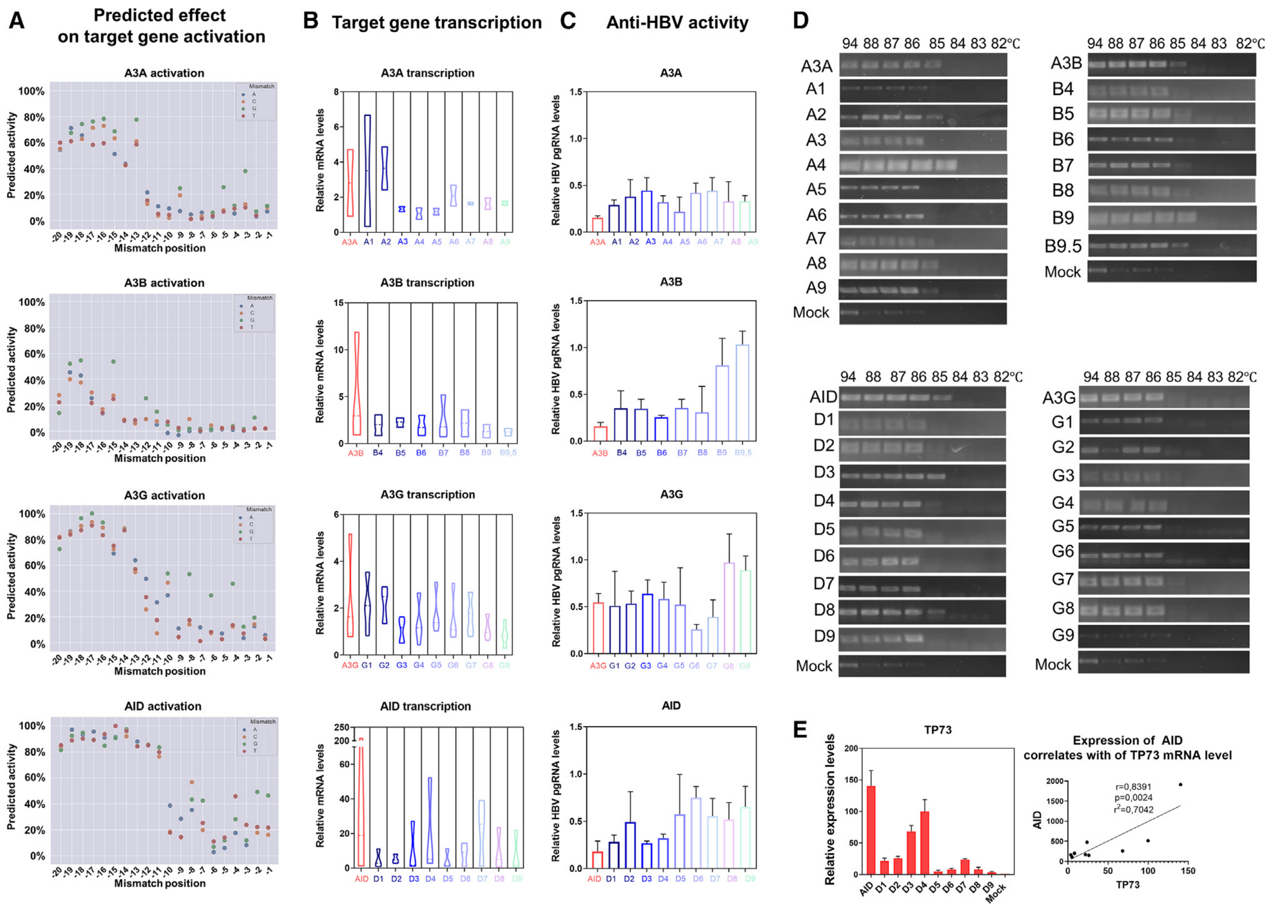

Figure 5. Titrating *APOBEC/AID* activation using att-sgRNAs and dCas9-p300

(A) Effect of single-nucleotide mismatches in specific positions (−20 to −1) of sgRNA on the activation of target genes, shown as % activation by sgRNA with no mismatches. (B) mRNA levels of target genes upon activation with original (A3A, A3B, A3G, AID) or att-sgRNAs (1–9 for *A3A*, *A3G*, and *AID*; 4–9.5 for *A3B*; numbers indicate 10% increments of predicted reduction in target gene activation, e.g., 1 means 10% predicted reduction, while 9.5 indicates 95% predicted reduction). (C) Anti-HBV activity of CRISPRa with original or att-sgRNAs measured by HBV transcription. Mock control (not shown) is set as 1. (D) 3D-PCR analysis of HBV cccDNA deamination in a CpG-rich region upon activation of APOBEC/AID genes with dCas9-p300 and original sgRNAs (A3A, A3B, AID, A3G) or att-sgRNAs. (E) Altered *TP73* expression upon CRISPR activation of the *AID* gene and correlation analysis of AID and TP73 mRNA levels. Target gene transcription, HBV pgRNA, and TP73 mRNA levels are normalized to GAPDH mRNA. Experiments were performed in the HepG2 rcccDNA transfection model. Mock, dCas9-p300 with a non-targeting sgRNA. The results represent the mean of at least three experiments ±SD.

To conclude, titrating activation levels of cytidine deaminases by CRISPRa allows APOBEC/AID proteins to retain antiviral properties even at low levels of upregulation, yet substantially diminishes the toxic effects of these overexpressed genes.

Att-sgRNAs eliminate off-site mutagenesis of CRISPRa RNPs while retaining robust anti-HBV activity

Using CRISPR-Cas RNPs, i.e., complexes of purified Cas proteins and *in vitro* synthesized sgRNAs, has emerged as an efficient method for introducing alterations into the genome. So far, only gene editing approaches have successfully managed to achieve long-term functional benefits.[47] Whether transient delivery of CRISPRa in the form of short-lived RNPs can be used in a therapeutic context is so far unclear. Hence, we next focused on exploring whether CRISPRa RNPs can suffice in suppressing HBV replication. We produced RNPs of a high-specificity *Streptococcus aureus* dCas9-p300 (dSaCas9) protein, which is considered safer compared with *Streptococcus pyogenes* dCas9 protein due to longer PAM and fewer off-target sites,[40] with *in vitro* transcribed sgRNAs targeting *A3A* and *A3B* genes, as these genes were less toxic than *AID* and *A3G*. A single transfection of dSaCas9 RNPs increased *A3A* and *A3B* transcription by ∼200- to 280-fold (adjusted to transfection efficiency) (Figures 6A and 6B) and resulted in a dramatic decline of HBV pgRNA (∼89%–90%), cccDNA (∼93%–98%), and HBsAg (∼52%–65%) levels (Figures 6C and 6D). 3D-PCR confirmed deamination of HBV cccDNA by CRISPRa RNPs in the remaining cccDNA (Figure 6E). Ultimately, for the first time, we demonstrate that transient activation of *A3A*

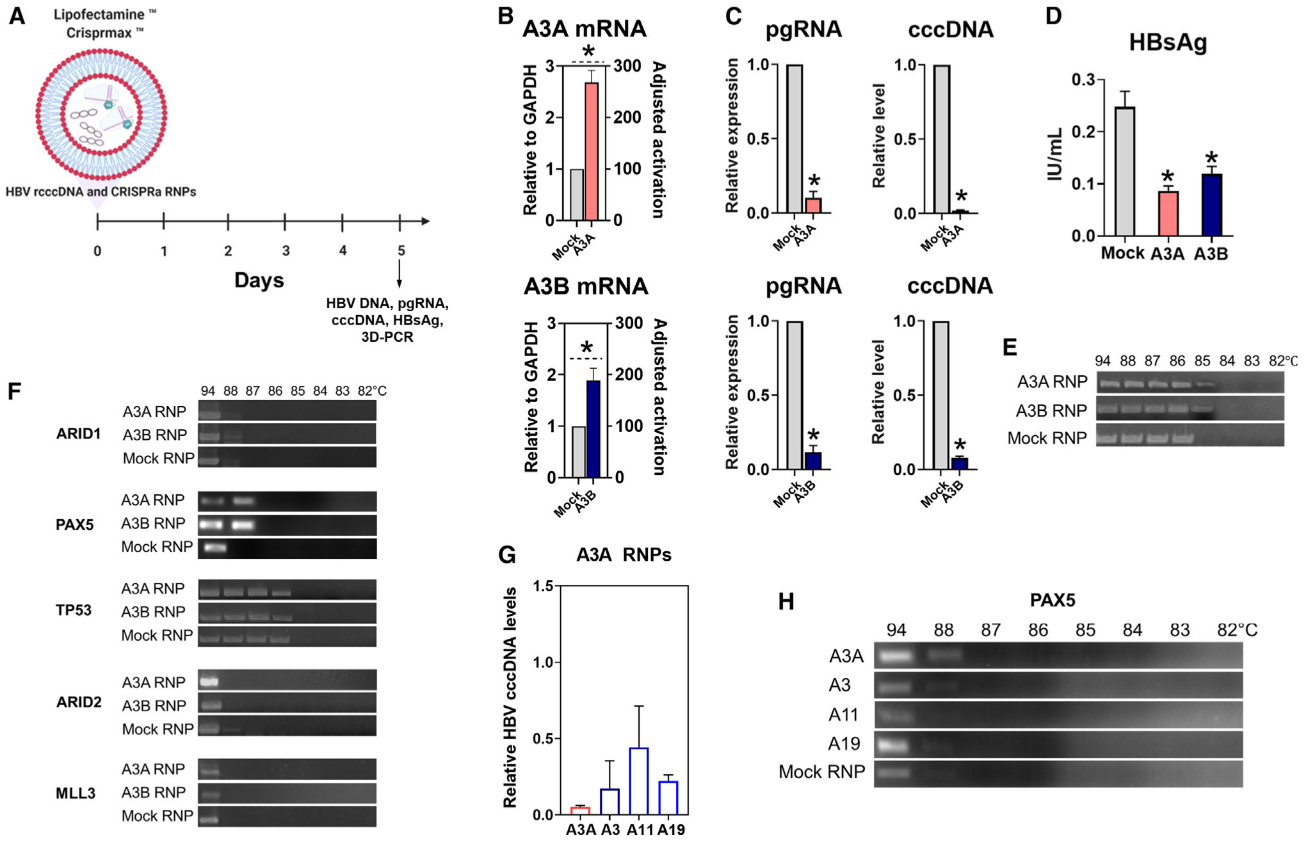

Figure 6. Effects of CRISPRa RNPs

(A) Experimental design of RNP transfection. (B) Activation of target genes by RNPs 24 h p.t. Anti-HBV activity of CRISPRa RNPs measured by (C) HBV pgRNA and cccDNA levels and (D) secreted HBsAg. (E) Analysis of HBV cccDNA deamination. (F) Off-target mutagenesis by CRISPRa RNPs. (G) Anti-HBV activity (measured by cccDNA levels) of CRISPRa RNPs targeting A3A gene with original (A3A) or att-sgRNAs (A3, A11, and A19 correspond to sgRNAs harboring mismatches at positions 3, 11, and 19, correspondingly). (H) Elimination of A3A off-target deamination at *PAX5* gene by attenuated sgRNAs. Mock RNP, dSaCas9-p300 protein with a non-targeting sgRNA. $^{\circ}p < 0.05$; $^{\Delta}p < 0.01$; $^{\#}p < 0.001$; $^{*}p < 0.0001$. The results represent the mean of at least three experiments ±SD. Figure 6A was created in BioRender.

and *A3B* by CRISPRa RNPs profoundly reduces HBV transcription, protein synthesis, and cccDNA levels.

The use of short-lived CRISPRa RNPs resulted in off-target deamination of *PAX5* gene upon activation of *A3A* (Figure 6F), similar to the use of CRISPRa plasmids (Figure 4C). In an attempt to eliminate off-site mutagenesis, we produced CRISPRa RNPs targeting the *A3A* gene with att-sgRNAs (A3, A11, A19 harboring mismatches at positions 3, 11, and 19, correspondingly). The use of att-sgRNAs retained pronounced anti-HBV activity (Figure 6G), but prominently reduced (for A3 and A19) or eliminated (for A11) deamination of *PAX5* gene (Figure 6H).

To conclude, attenuated sgRNA can be used to fine-tune CRISPRa activity and eliminate/reduce potential side effects of activated genes. Overall, the described approach represents a novel antiviral strategy, for the first time demonstrating the feasibility of using CRISPRa RNPs and attenuated sgRNA technology as antiviral and safety control tools.

Activity of CRISPRa RNPs at the models of established HBV infection

Next, we tested CRISPRa RNPs with original (A3A) and att-sgRNAs at advanced *in vitro* models of established HBV infection, including HepG2-hNTCP (NTCP-complemented HepG2 cells supporting HBV infection) and differentiated HepaRG-hNTCP (non-transformed NTCP-overexpressing cells forming hepatocyte islands) cell lines (Figure 7). Cells were infected with HBV, and nucleofected with CRISPRa RNPs 7 days after. CRISPRa RNPs resulted in similar levels of A3A activation (6- to 11-fold) between the cell lines with evident attenuation of A3A activation using att-sgRNAs (Figures 7A and 7B). Analysis of antiviral activity 6 days post CRISPRa RNPs nucleofection revealed that HBsAg was not reduced by CRISPRa RNPs (Figures 7C and 7D), while HBeAg (a surrogate marker of cccDNA) was significantly reduced only in HepaRG-hNTCP cells (Figure 7D). HBV cccDNA and pgRNA levels dropped by over 60%–80% with comparable reduction rates between original and attenuated sgRNAs (Figures 7C and 7D). The non-impressive reduction in viral antigen levels

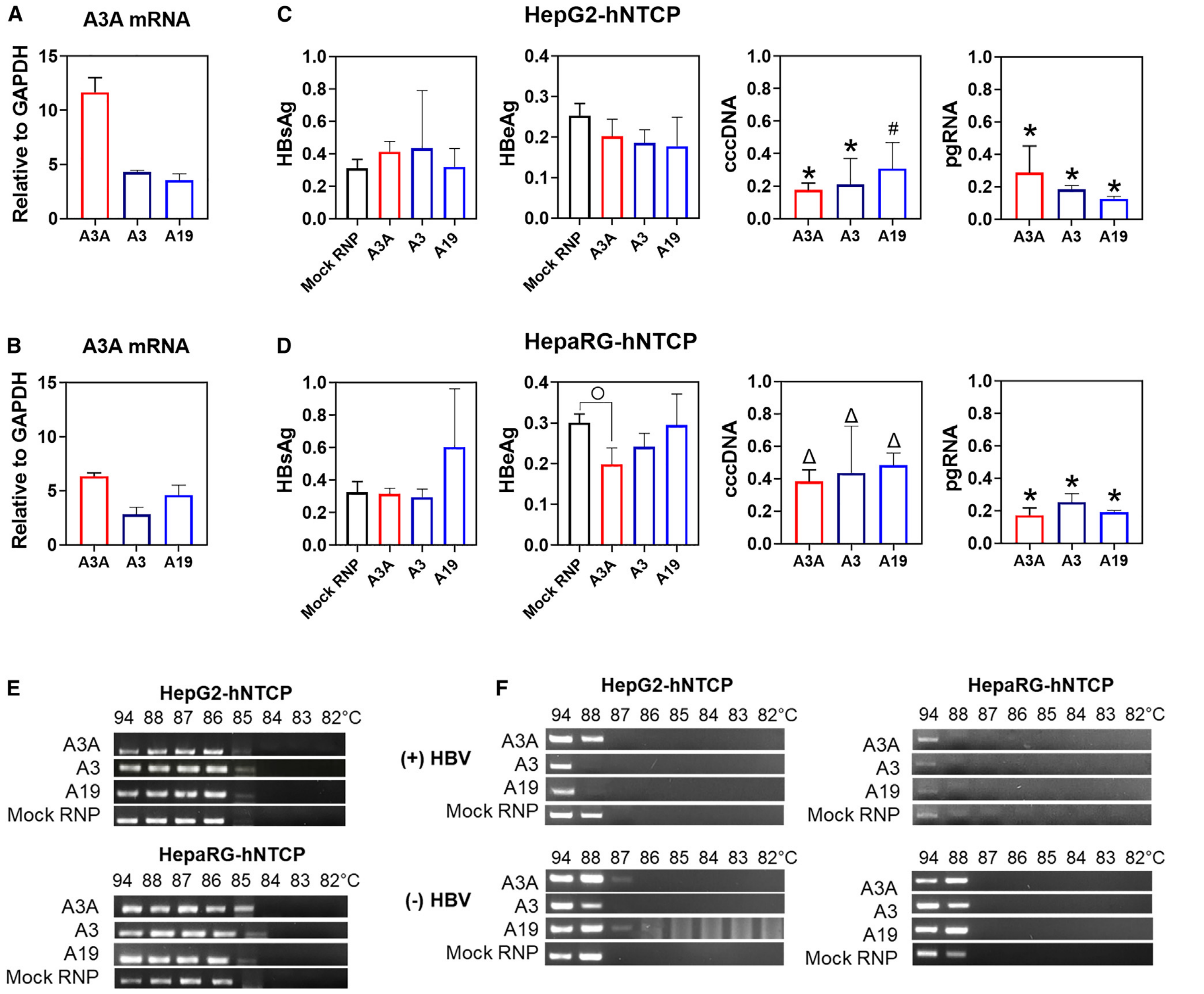

Figure 7. CRISPR activation of A3A at HBV infection models

CRISPR activation of A3A at (A) HepG2-hNTCP and (B) HepaRG-hNTCP cell lines. Anti-HBV activity at HBV-infected (C) HepG2-hNTCP and (D) HepaRG-hNTCP cells. A3A mRNA levels were measured 20 h post RNPs nucleofection. CRISPRa RNPs were nucleofected 7 days post HBV infection; anti-HBV activity was measured 6 days post nucleofection. Original sgRNA (A3A) and att-sgRNAs (A3 and A19) were used in a complex with dSaCas9-p300. (E) On-target HBV cccDNA deamination the first day post nucleofection. (F) Off-target 3D-PCR analysis of *PAX5* gene the first day post nucleofection. Off-site analysis was performed in (+) HBV (HBV-infected) and (–) HBV (non-infected) cell lines. Mock RNP, dSaCas9-p300 protein with a non-targeting sgRNA. $^{\circ}p < 0.05$; $^{\Delta}p < 0.01$; $^{\#}p < 0.001$; $^{*}p < 0.0001$.

contrastingly to cccDNA and pgRNA at the models of established infection may be explained by relatively long half-lives of HBV antigens (over 6 days for HBsAg)[48] and re-establishment of HBV replication following short-term exposure to antivirals.[8] Strong suppression of HBsAg secretion in rcccDNA experiments (Figure 6D) is thus likely related to rapid inactivation of rcccDNA and prevention of HBsAg production.

On-target 3D-PCR of cccDNA demonstrated deamination similar to previous experiments (Figure 7E). Off-site deamination at the *PAX5* gene was investigated in HBV-infected and non-infected cells (Figure 7F). We did not observe *PAX5* deamination in cells infected with HBV, whereas in uninfected cells *PAX5* was noticeably deaminated both in HepG2-hNTCP (A3A and A19 groups) and HepaRG-hNTCP cells (A3A, A3, and A19 groups). Therefore, HBV infection indeed protects the genome from genotoxic effects of A3A to a certain extent (Figure 7D).

To conclude, we proved the potent antiviral activity of transiently CRISPR-activated A3A at models of established HBV infection

in vitro and demonstrated comparable anti-HBV activity of CRISPRa RNPs with attenuated sgRNAs.

DISCUSSION

CRISPR-based technologies are being actively investigated for use in clinical applications. CRISPR nucleases, base editors, and prime editors applicable for treatment of hereditary diseases, cancers, and HIV infection have recently progressed to the early phases of clinical trials.[47] However, for many practical reasons, the use of these tools is challenging. The issue of irreversible off-target activity and toxicity limits the use of CRISPR nucleases and base editors.[49] The use of CRISPRa-based therapies is advantageous due to reversible activation of genes of interest, low risk of off-target activity, and ability to modulate multiple disease-associated parameters at the same time.[33] CRISPRa tools have been successfully used to instigate antiviral[50] immunity and correct hereditary diseases.[28,40]

Similarly, substantial progress has been made in using CRISPR[51] technologies for inactivating HBV cccDNA. Many CRISPR-Cas nucleases were used to effectively target and destroy HBV cccDNA.[52,53] However, one of the major challenges in the use of Cas nucleases for eliminating HBV from infected cells is nucleolytic cleavage of HBV genome integrations, which may lead to cell death or, in the worst scenario, to oncotransformation and development of cancer.[54] APOBEC/AID factors can also potentially target HBV DNA genomic integrations, but this might rather result in mutational inactivation of viral integrations and would not result in highly pernicious, DSBs. Here, we provide the findings of using CRISPRa for modulating expression of APOBEC/AID factors and eliminating HBV from cells. Compared with CRISPR-Cas nucleases, this approach does not cleave viral DNA and does not induce harmful DNA double-strand breaks. Moreover, off-target activity is not an issue for CRISPRa, as off-site epigenetic modifications or their effects on transcription of non-targeted genes were not detected. Importantly, this study also first demonstrates that transient CRISPR activation of *APOBEC/AID* genes in the form of RNPs achieves >60%–98% reduction in HBV replication (Figures 2 and 6). The extent of HBV cccDNA deamination and reduction in HBeAg levels induced by high doses of interferon-α (activator of A3A) and lymphotoxin-β-receptor agonist (activator A3B) were more pronounced in a previous study.[18] This is likely related to very transient activation of APOBEC/AID achieved in our experimental setup or by additional co-factors activated by interferons/lymphotoxin-β-receptor agonist which might be responsible for additional suppression of viral replication[55] and degradation of cccDNA.[38]

Transcriptomic profiling of HBV cells with CRISPRa demonstrated similar changes in the expression of DDR factors for A3A, A3B, and AID groups (activation of *EXO1*, *MPG*, and *XRCC3*) (Figure 3B). Still, we observed unique transcriptomic alterations for each cytidine deaminase, suggesting differences in outcomes and mechanisms of the cellular responses to deamination. The transcriptomic profile of the A3G group was the most distinct from those of the other deaminases, with *EXO1* being the single gene commonly upregulated by all deaminases used (Figure 3B). Collectively, these transcriptomic changes indicate signaling pathways responsible for HBV cccDNA mutation repair and degradation.

So far, CRISPRa approaches have been used for prolonged overexpression of target genes, which is not clinically relevant. For the first time, we demonstrate prominent anti-HBV activity of short-lived CRISPRa RNPs (Figure 6) that function for up to 24 h and then decay. Taken together, our data highlight the prospects of using CRISPRa RNPs for modulating antiviral genes and, potentially, treating HBV infection. The issues of CRISPRa immunogenicity, dosing, and delivery will have to be resolved for *in vivo* applications. In this respect, different nanoparticles are promising delivery vehicles for CRISPRa RNPs.[56] However, delivering activators of APOBEC/AID proteins into HBV-infected cells is mandatory for ensuring a good safety profile of potential drug candidates.

Although APOBEC/AID are famous mutators implicated in carcinogenesis and DNA damage,[22] several studies have shown that the viral HBcAg protein may shield the host genome and target APOBEC/AID enzymes directly to HBV cccDNA.[18,44] Thus, APOBEC/AID enzymes may be used for developing treatment strategies for HBV infection. In this study, we demonstrate that mutagenic properties of APOBEC/AID may indeed be restrained by the virus, but only during active viral replication. When viral loads are low, APOBEC/AID induced off-target deamination in cancer-associated genes (Figure 4). The threshold of viral loads that are protective to the genome and the therapeutic window of cytidine deaminase doses must be defined in further studies. It is likely that APOBEC/AID-induced mutations may not be detected due to low coverage of specific genes (>30 in previous studies[45]), which may be insufficient (3D-PCR amplicons have a coverage of >30,000–100,000 in our study). Using short-lived CRISPRa RNPs and att-sgRNAs, we are the first to show the opportunity for mitigating toxicity and pro-mutagenic effects of *APOBEC/AID* while retaining these enzymes' potent antiviral activity.

To conclude, here we demonstrate for the first time the proof-of-principle for using CRISPRa to induce anti-HBV immunity and eliminate HBV cccDNA from cells. Coupling CRISPRa RNPs with att-sgRNA technology[30] for simultaneous, tunable, and transient regulation of complex antiviral programs paves the way for using CRISPRa approaches as a promising antiviral strategy. The described results can be used for developing novel therapeutic and, potentially, curing strategies for other infectious diseases.

MATERIALS AND METHODS

Cell culture and transfection

All cell lines (HepG2, HepG2-1.1merHBV, HepG2-1.5merHBV) were cultured in DMEM high-glucose medium with 10% fetal bovine serum, 2 μM L-glutamine, and 1% penicillin/streptomycin. HepG2 cells were transfected with dCas9-p300-encoding plasmid (pcDNA-dCas9-p300 Core or a mutant form with an inactivated p300 acetyltransferase pcDNA-dCas9-p300 Core [D1399Y]), U6-PCR product encoding sgRNA, and HBV rcccDNA produced using minicircle

technology (described in Kostyushev et al.[52,57] and Li et al.[57]). After 48 h, cell culture medium was discarded and cells were washed twice in PBS and cultured in new complete medium for the next 72 h. Similarly, HepG2-1.1merHBV and HepG2-1.5merHBV cell lines (kindly provided by Dr. Dieter Glebe) were transfected using Lipofectamine 3000 with dCas9-p300/Blast-encoding plasmid and PCR product. Doxycycline was added to HepG2-1.1merHBV cells for 24 h to induce viral replication, then washed with PBS. Transfection of HepG2-1.1merHBV was performed 24 h after adding doxycycline. All results were reproduced in at least three independent studies.

Synthesis of recombinant HBV cccDNA

Recombinant HBV cccDNA was generated using minicircle technology (System Biosciences). Briefly, the genome of HBV genotype D was cloned into a minicircle-producing plasmid with attB and attP recombination sites (mini-HBV). *E. coli* strain ZYCY10P3S2T (System Biosciences) was transformed with mini-HBV, and clones were selected and incubated for 4 h at 37°C in Luria broth (LB) containing kanamycin. The resulting cell suspension was inoculated into 200 mL of TB media and incubated overnight, mixed with 200 mL of induction media (1 N NaOH and 0.2% L-arabinose in LB), and incubated for 3 h at 30°C and then for 1 h at 37°C. HBV rcccDNA was isolated from the resulting bacterial pellet using a QIAGEN Plasmid Maxi Kit (Qiagen).

CRISPR-Cas9 constructs

sgRNAs targeting promoters of genes of interest (*A3A*, *A3B*, *A3G*, *AID*) were designed using the open-access web tool Broad Institute Genetic Perturbation Platform. PCR products containing the U6 promoter and sgRNA specific for every promoter region were synthesized by two-step mutagenic PCR using Q5 polymerase and purified using a Qiagen gel extraction kit.[52,53] The following plasmids were used: pcDNA-dCas9-p300 Core (AddGene plasmid #61357[23]) and pcDNA-dCas9-p300 Core (D1399Y), gifts from Dr. Charles Gersbach; pLX-sgRNA (AddGene plasmid #50662[58]), a gift from Dr. Eric Lander and Dr. David Sabatini; dCas9-p300 Core was cloned into Lenti-Cas9-2A-Blast plasmid (AddGene plasmid #73310,[59] a gift from Dr. Jason Moffat) instead of *Streptococcus pyogenes* Cas9. dSaCas9-p300 was generated from pJEP303-pAAV-CMV-dSaCas9-VP64-pA (AddGene plasmid #113678,[60] a gift from Dr. Jonathan Ploski) by cloning dSaCas9 into pcDNA-dCas9-p300 Core plasmid. dSaCas9 sgRNAs were produced from a pSaGuide (Addgene plasmid #64710; a gift from Dr. Kiran Musunuru). All primers and probes are listed in Table S19.

Production of dSaCas9-p300 protein

The protein was produced in *E. coli* strain BL21 (DE3) pLysS cells (Novagen). Cells were grown in LB medium supplemented with the appropriate antibiotic, 0.5% sucrose, 0.5% glycerol, 1 mM magnesium chloride, 50 mM di-sodium hydrogen phosphate, 50 mM potassium dihydrogen phosphate, and 25 mM ammonium sulfate, and grown at 30°C to OD_{600} of 1.2. Protein expression was induced by incubation with 0.1 mM IPTG for 16 h at 18°C. dSaCas9-p300 protein was purified by a combination of affinity and ion-exchange chromatography steps. Cells were resuspended in 50 mM Tris/HCl (pH 8.0), 500 mM NaCl, 1 mM PMSF, 0.2% Triton X-100, and 0.1% Tween 20, sonicated on ice, and centrifuged at 15,000 × *g* for 40 min. The lysate was treated with 0.05% polyetheleneimine for 30 min at 4°C; the resulting suspension was centrifuged at 15,000 × *g* for 40 min. Supernatant was bound to Ni-Chelating Sepharose (GE Healthcare). The resin was washed extensively with 50 mM Tris/HCl (pH 8.0), 500 mM NaCl, and 0.05% Igepal CA-630, and bound protein was eluted in 50 mM Tris/HCl (pH 8.0), 150 mM NaCl, 0.3 M imidazole, and 10% glycerol. The protein was further bound to SP Sepharose (GE Healthcare) in 50 mM Tris/HCl (pH 7.5), 150 mM NaCl, 0.01% Triton X-100, and 2 mM DTT, and eluted with a linear gradient of 150 mM to 1 M NaCl. Glycerol was added to the final concentration of 50%; protein was stored at −20°C.

In vitro sgRNA transcription and purification

PCR products encoding the appropriate sgRNA sequence under control of a T7 promoter were synthesized using Q5 polymerase (NEB). T7-containing PCR products were used for *in vitro* transcription (IVT) reaction using HiScribe Quick T7 High Yield RNA synthesis kit (NEB) according to the manufacturer's protocol. After overnight incubation, the IVT reaction was treated with DNAse I (NEB) for 15 min at 37°C followed by isopropanol purification. Air-dried pellets were resuspended in RNase-free water and stored at −80°C.

Generation and transfection of dSaCas9-p300 ribonucleoprotein complexes

Recombinant dSaCas9-p300 protein was mixed with IVT sgRNAs at a 1:1 M ratio in OptiMEM (Gibco) and incubated for 10 min to assemble RNPs, followed by addition of 300 ng of target rcccDNA. HepG2 cells were transfected with the mix of RNPs and rcccDNA using a Lipofectamine CRISPRMAX transfection kit (Invitrogen). Briefly, Cas9 Plus reagent was added to the mix of RNPs and rcccDNA, mixed, and incubated for 10 min. In parallel, CRISPRMAX reagent was mixed with OptiMEM (Gibco) and incubated for 10 min. Next, CRISPRMAX mix was added to the RNPs/rcccDNA complexes and incubated for another 10 min. The final mixture was added to cells at 50% of confluence.

Isolation of nucleic acids

At harvest, cell culture medium was discarded, and cells were washed twice with PBS and lysed in AmpliSens Riboprep lysis buffer (AmpliSense Biotechnologies). Nucleic acids were isolated using the AmpliSense Riboprep kit (AmpliSense Biotechnologies) according to the manufacturer's instructions. To isolate RNAs, nucleic acids were treated with RNase-free DNase I (NEB) for 30 min at 37°C, purified using the AmpliSense Riboprep kit (AmpliSense Biotechnologies), and reverse-transcribed using AmpliSens Reverta-FL (AmpliSense Biotechnologies). HBV cccDNA was isolated following the Hirt procedure as described by Cai et al.,[61] followed by treatment with plasmid-safe ATP-dependent DNase (Epicentre) for 12 h at 37°C and inactivation of enzyme at 72°C for 15 min.

PCR analysis

Expression of cellular mRNAs, HBV pgRNA, and S-mRNA was normalized to GAPDH mRNA levels. Total intracellular HBV

DNA and cccDNA levels were normalized to levels of genomic β-globin. All PCRs were performed with specific sets of primers and probes (Table S19) using real-time PCR cyclers Rotor-Gene 6000 (Corbett Research) and CFX96 cycler (BioRad). Relative expression levels were calculated by the $^{\Delta\Delta}$Ct method.

ChIP-PCR

HepG2 cells were seeded into 150-mm Petri dishes at 50% confluence and transfected using Lipofectamine 3000 with HBV rcccDNA, dCas9-p300-expressing plasmid, and a PCR product encoding a corresponding sgRNA. Forty-eight hours post transfection, cells were washed with PBS, harvested using trypsin, and counted; 9×10^6 cells were used per single ChIP reaction. Cells were fixed in 1% paraformaldehyde and lysed using ChIP Kit (ab500, Abcam) reagents. Chromatin was degraded into 200- to 1,000-nt fragments using the ultrasound disintegrator Branson Sonifier 250. An aliquot of fragmented chromatin was frozen at −80°C for PCR normalization of test results. After that, chromatin fragments were incubated with anti-acetyl histone 3 (acH3) (06–599; Merck) antibodies overnight at 4°C; incubation with anti-histone 3 (ab1791; Abcam) antibodies served as a positive control. Bound chromatin was isolated using affinity purification beads (ab500, Abcam). Semi-quantitative PCR analysis with primers amplifying regions of *APOBEC/AID* promoters was used for assessing enrichment of acH3 at CRISPRa-targeted regions. ChIP-PCR primers were designed using the UCSC Genome Browser and Primer-BLAST online tool. ChIP-PCR primers are listed in Table S19.

Microarray assay

Total mRNA was isolated using the RNeasy Mini Kit (Qiagen). cDNA was prepared using the RT2 PreAMP cDNA Synthesis Kit (Qiagen) and PCR-amplified with RT2 SYBR Green qPCR Mastermixes (Qiagen). Alterations in DNA damage signaling were assessed using RT2 Profiler PCR Array Human DNA Damage Signaling Pathway on a Real-Time CFX96 cycler (BioRad) according to the manufacturer's instructions.

Cell viability and proliferation

Cells were seeded onto 96-well plates to 70% confluence and transfected using Lipofectamine 3000. Proliferation and viability of cells were measured 0–4 days p.t. using the Cell Cytotoxicity Assay (ab112118) according to the manufacturer's protocol. Colorimetric signals were detected by iMark Microplate Absorbance Reader (BioRad).

Immunofluorescence

Cells were seeded onto glass coverslips, transfected using Lipofectamine 3000, and fixed in 4% paraformaldehyde for 10 min before harvesting. Next, coverslips were washed three times in Tris-HCl (50 mM, pH 8.0) and incubated for 30 min with blocking buffer (0.02% Triton X-100, 10% horse serum, and 150 mM NaCl in Tris-HCl [50 mM, pH 8.0]). The coverslips were then incubated with primary rabbit anti-HBc antibodies (ab115192) or rabbit anti-53BP1 antibodies (ab21083) and mouse γ-H2AX antibodies (ab26350) at room temperature for 1 h, washed three times for 5 min in washing buffer (0.02% Triton X-100 and 200 mM NaCl in Tris-HCl [50 mM, pH 8.0]), and incubated with secondary Alexa Fluor 488 goat anti-rabbit immunoglobulin (Ig)G antibodies (ab150077) and Alexa Fluor 594 goat anti-mouse IgG H & L antibodies (ab150116) with Hoechst33342 (1:10,000) at room temperature for 1 h. Alternatively, cells were stained with anti-A3A (Abcam ab38641), anti-A3B (ab104759), anti-A3G (ab194581), or anti-AID (ab59361) antibodies. Coverslips were washed three times for 5 min in washing buffer and mounted with Fluoroshield reagent (ab104135). Images were captured on a Leica DMI6000 microscope at ×20 and ×63 immersion objective.

Western blotting

Transfected HepG2 cells were harvested from six-well plates, lysed on ice with 100 μL of ice-cold RIPA buffer for 10 min, mixed with 100 μL of Laemmle buffer, and kept on ice for 10 min. Next, samples were heated at 95°C for 10 min. Samples were stored at −20°C until use. Proteins were resolved by SDS-PAGE electrophoresis and transferred to PVDF Hybond-P (Amersham GE healthcare) membranes. Membranes were blocked with 5% fat-free powdered milk in PBS-T buffer (80 mM Na_2HPO_4, 20 mM NaH_2PO_4, 100 mM NaCl, 0.1% Tween 20) at room temperature for 1 h or at 4°C overnight. After that, membranes were incubated with primary anti-A3A (Abcam ab38641), anti-A3B (ab104759), anti-A3G (ab194581), or anti-AID (ab59361) antibodies, or with rabbit anti-53BP1 antibodies (ab21083), mouse γ-H2AX antibodies (ab26350), or rabbit anti-H2AX antibodies (ab11175) at 4°C overnight with gentle shaking. Samples were then washed three times for 10 min with PBS-T and incubated with secondary Goat Anti-Rabbit IgG H&L (HRP) (ab205718, diluted to 1:10,000) antibodies or secondary goat anti-mouse IgG (ab6789, diluted to 1:10,000) antibodies conjugated to horseradish peroxidase. Finally, membranes were washed three times with PBS-T; signal was developed with enhanced chemiluminescence reagent (Thermo Fisher Scientific) and detected with G:box Chemi XX6 (Syngene) using Genesis software (Syngene) or using X-ray films. Then, membranes were stripped using mild stripping buffer (1.5% glycine, 0.1% SDS, and 1% Tween 20 [pH 2.2]) and re-stained with primary mouse anti-β-actin (Sigma, A5441, 1:10 000) monoclonal antibodies or anti-α-tubulin (Sigma, T6199, 1:10 000) for 1 h at room temperature. To detect signal, samples were washed three times with PBS-T and incubated with secondary goat anti-mouse IgG (ab6789, 1:10,000) antibodies conjugated to horseradish peroxidase. Results were analyzed using ImageJ software.

3D-PCR and NGS

CpG-rich regions of HBV cccDNA or genomic DNA were amplified with a pair of specific primers using TaqF polymerase; amplicons were gel-purified and extracted using Qiagen gel extraction kit. Equal amounts of purified PCR products were used for nested PCR with TaqF polymerase at decreasing temperatures (95°C–82°C) and then used for gel electrophoresis and next-generation sequencing (NGS). Alternatively, 3D-PCR was performed semi-quantitatively with SYBRGreen dye. In brief, 3D-PCR amplicons obtained at 87°C, 84°C, or 82°C were gel-purified and extracted using a Qiagen gel extraction kit, quantified with a Qubit 2.0 Fluorometer (Life

Technologies), and pooled in equimolar ratios. Adapters for Illumina sequencing were then attached. Libraries were sequenced with 250 paired-end reads using MiSeq instrument (Illumina). FASTQC software and Geneious software were used for assessing quality, aligning references, discarding low-quality reads and nucleotides, and calculating indels. Custom Python codes (available upon request) were used for mutation analysis and mutation context analysis. 3D-PCR primers are listed in Table S18.

Comet assay

Potential genotoxicity was measured using the Comet SCGE assay kit (Enzo Life Sciences). In brief, HepG2 or HepG2-1.5merHBV cell lines were harvested 4 or 5 days post transfection; 2,000 cells were resuspended in melted LTM agarose at 37°C, and 75 μL of the suspension was layered onto prepared glass slips and left to solidify at 40°C. Next, the glass slips were incubated in lysis buffer for 45 min and then moved into alkaline buffer (pH > 13) for 50 min. The slips were washed twice for 5 min in TBE buffer and then transferred into an electrophoresis chamber with TBE buffer (1 V/cm for 50 min). After electrophoresis, the slips were merged into 70% ethanol twice for 5 min and then air-dried. Cell nuclei on the slips were counterstained by SYBRGreen for 30 min, washed twice in deionized water, and dried at 37°C. Visualization of comets was performed using the FITC channel of an Olympus IX71 fluorescent microscope.

Experiments with attenuated sgRNAs

Attenuated sgRNAs were designed using an algorithm described by Jost et al.[30] Synthesis of each sgRNA was performed as described previously[62,63] using a two-step mutagenic PCR. Attenuated sgRNAs with dCas9-p300-expressing plasmid and rcccDNA were transfected into HepG2 cells using Lipofectamine 3000. Original sgRNA targeting APOBEC/AID promoters and non-targeting sgRNA served as positive and negative controls, correspondingly.

HBsAg and HBeAg analysis

Cell-conditioned media were harvested and filtered through 0.2-μm filters to remove cell debris. HBsAg quantification was performed using the colorimetric ELISA test DS-IFAHBsAg-0.01 according to the manufacturer's protocol (Diagnostic Systems, Russia); HBeAg quantification was performed using colorimetric ELISA test DS-IFA-HBeAg (Diagnostic Systems, Russia).

Infection of HepG2-hNTCP and HepaRG-hNTCP cells

HepaRG-hNTPC cells were cultured in Williams E medium (Thermo) supplemented with 10% FetalClone II, Glutamax (Thermo), 5 μg/mL insulin, 50 μM hydrocortisone, 100 U/mL penicillin, and 100 μg/mL streptomycin. Cells were seeded at a density of 2×10^6 cells per dish (TPP, Switzerland) and grown to 90% density. Hepatitis B virus stock was obtained from the HepG2.2.15 cells. Cells were seeded into 10-cm dishes (TRP, Switzerland) precoated with type I rat collagen in DMEM containing 10% fetal serum. The cells were grown without splitting for 3 days after reaching monolayer with subsequent replacement of the medium with DMEM-F12 medium supplemented with 2.5% FetalClone II and 2% DMSO. The conditioned medium containing virions was collected every 3 days during 2 weeks. HEPES (pH 7.4) was added to the medium to 10 mM concentration before storage at +4°C. All aliquots were pooled, filtered through a 0.22-μm filter, the virions were harvested by precipitation by 8% PEG-8000 during gentle shaking at +4°C for 24 h with subsequent centrifugation (4,000 rpm, 1 h, +4°C). The pellet from 20 cell dishes was resuspended in 15 mL DMEM-F12 supplemented with 2.5% FetalClone II and stored in 0.5-mL aliquots at −80°C. Virus titer was determined by measuring the number of genomic equivalents, as described in Alfaiate et al.[63] HepG2-hNTPC or HepaRG-hNTCP cells were seeded on 12-well plates at a density of 1×10^5 cells/well. After reaching the monolayer, the cells were kept for 4 days without splitting. After addition of DMSO to 1.8% concentration, the cells were incubated for an additional 3 days, and then tetracycline was added to a concentration of 1 μg/mL. Twenty-four hours later, the cells were infected with HBV as described in Alfaiate et al.[63]

Statistical analysis

Values were expressed as the mean ± SD of triplicate experiments using SPSS software (SPSS 21.0.0.0). One-way ANOVA and Student's t tests with Tukey's HSD post hoc tests were used to compare variables and calculate p values to determine statistically significant differences in means.

DATA AVAILABILITY STATEMENT

All data are available upon a reasonable request.

SUPPLEMENTAL INFORMATION

Supplemental information can be found online at https://doi.org/10.1016/j.omtn.2023.04.016.

ACKNOWLEDGMENTS

This study was supported by RSF grant no. 22-75-10032 with the exception of experiments with HepG2-hNTPC and HepaRG-hNTCP cells, which were supported by the Russian Ministry of Science and Higher Education of the Russian Federation (agreement 075-15-2019-1660). Section with the design and initial testing of att-sgRNAs was supported by RFBR grant no. 20-515-12010. We cordially thank Dr. Dieter Glebe for providing HepG2-1.1merHBV and HepG2-1.5merHBV cell lines, and for his constant help and support at all stages of the project.

AUTHOR CONTRIBUTIONS

Conceptualization, D.K., S.B., A. Kostyusheva; methodology, D.K., A. Kostyusheva, S.B., N.P., E.B., A.S., I. Goptar, A.N., N.Z., and A. Kondrashova; investigation, D.K., S.B., A. Kostyusheva, N.Z.; data curation, D.K., S.B., A. Kostyusheva, E.B., N.P.; data analysis, D.K., S.B., A. Kostyusheva, N.P.; resources, V.C., I. Gordeychuk, A.L., A.I., A.Z.; writing – original draft, D.K.; writing – review and editing, D.K., A. Kostyusheva, S.B., E.B., I. Gordeychuk, A.L., A.Z., V.C.; supervision, D.K., V.C.; project administration, D.K., V.C.; funding acquisition, A.I., D.K., V.C.

DECLARATION OF INTERESTS

The authors declare no competing interests.

REFERENCES

1. World Health Organization (2017). Global Hepatitis Report.
2. Werle–Lapostolle, B., Bowden, S., Locarnini, S., Wursthorn, K., Petersen, J., Lau, G., Trepo, C., Marcellin, P., Goodman, Z., Delaney, W.E., 4th, et al. (2004). Persistence of cccDNA during the natural history of chronic hepatitis B and decline during adefovir dipivoxil therapy1. Gastroenterology *126*, 1750–1758.
3. Kostyusheva, A., Brezgin, S., Glebe, D., Kostyushev, D., and Chulanov, V. (2021). Host-cell interactions in HBV infection and pathogenesis: the emerging role of m6A modification. Emerg. Microbes Infect. *10*, 2264–2275. https://doi.org/10.1080/22221751.2021.2006580.
4. Nassal, M. (2015). HBV cccDNA: viral persistence reservoir and key obstacle for a cure of chronic hepatitis B. Gut *64*, 1972–1984. https://doi.org/10.1136/gutjnl-2015-309809.
5. Volz, T., Allweiss, L., Ben MBarek, M., Warlich, M., Lohse, A.W., Pollok, J.M., Alexandrov, A., Urban, S., Petersen, J., Lütgehetmann, M., and Dandri, M. (2013). The entry inhibitor Myrcludex-B efficiently blocks intrahepatic virus spreading in humanized mice previously infected with hepatitis B virus. J. Hepatol. *58*, 861–867. https://doi.org/10.1016/j.jhep.2012.12.008.
6. Tu, T., Zehnder, B., Qu, B., and Urban, S. (2021). D e novo synthesis of hepatitis B virus nucleocapsids is dispensable for the maintenance and transcriptional regulation of cccDNA. JHEP Rep. *3*, 100195. https://doi.org/10.1016/j.jhepr.2020.100195.
7. Ko, C., Chakraborty, A., Chou, W.-M., Hasreiter, J., Wettengel, J.M., Stadler, D., Bester, R., Asen, T., Zhang, K., Wisskirchen, K., et al. (2018). Hepatitis B virus (HBV) genome recycling and de novo secondary infection events maintain stable cccDNA levels. J. Hepatol. *69*, 1231–1241.
8. Kostyushev, D., Kostyusheva, A., Brezgin, S., Ponomareva, N., Zakirova, N.F., Egorshina, A., Yanvarev, D.V., Bayurova, E., Sudina, A., Goptar, I., et al. (2023). Depleting hepatitis B virus relaxed circular DNA is necessary for resolution of infection by CRISPR/Cas9. Mol Ther Acids *31*, 482–493.
9. Wedemeyer, H., Schöneweis, K., Bogomolov, P.O., Voronkova, N., Chulanov, V., Stepanova, T., Bremer, B., Allweiss, L., Dandri, M., Burhenne, J., et al. (2019). Final results of a multicenter, open-label phase 2 clinical trial (MYR203) to assess safety and efficacy of myrcludex B in combination with PEG-interferon Alpha 2a in patients with chronic HBV/HDV co-infection. J. Hepatol. *70*, e81.
10. Kostyusheva, A., Kostyushev, D., Brezgin, S., Volchkova, E., and Chulanov, V. (2018). Clinical implications of hepatitis B virus RNA and covalently closed circular DNA in monitoring patients with chronic hepatitis B today with a gaze into the future: the field is unprepared for a sterilizing cure. Genes *9*, 483. https://doi.org/10.3390/genes9100483.
11. Feng, J., Wickenhagen, A., Turnbull, M.L., Rezelj, V.V., Kreher, F., Tilston-Lunel, N.L., Slack, G.S., Brennan, B., Koudriakova, E., Shaw, A.E., et al. (2018). Interferon-stimulated gene (ISG)-Expression screening reveals the specific antibunyaviral activity of ISG20. Jung JU. J. Virol. *92*, e02140-17. https://doi.org/10.1128/JVI.02140-17.
12. Stavrou, S., and Ross, S.R. (2015). APOBEC3 proteins in viral immunity. J. Immunol. *195*, 4565–4570. https://doi.org/10.4049/jimmunol.1501504.
13. Moris, A., Murray, S., and Cardinaud, S. (2014). AID and APOBECs span the gap between innate and adaptive immunity. Front. Microbiol. *5*, 534.
14. Okada, A., and Iwatani, Y. (2016). APOBEC3G-Mediated G-to-A hypermutation of the HIV-1 genome: the missing link in antiviral molecular mechanisms. Front. Microbiol. *7*, 2027. https://doi.org/10.3389/fmicb.2016.02027.
15. Stavrou, S., Nitta, T., Kotla, S., Ha, D., Nagashima, K., Rein, A.R., Fan, H., and Ross, S.R. (2013). Murine leukemia virus glycosylated Gag blocks apolipoprotein B editing complex 3 and cytosolic sensor access to the reverse transcription complex. Proc. Natl. Acad. Sci. USA *110*, 9078–9083. https://doi.org/10.1073/pnas.1217399110.
16. Zhang, Y., Chen, X., Cao, Y., and Yang, Z. (2021). Roles of APOBEC3 in hepatitis B virus (HBV) infection and hepatocarcinogenesis. Bioengineered *12*, 2074–2086. https://doi.org/10.1080/21655979.2021.1931640.
17. Nair, S., and Zlotnick, A. (2018). Asymmetric modification of hepatitis B virus (HBV) genomes by an endogenous cytidine deaminase inside HBV cores informs a model of reverse transcription. J. Virol. *92*, e02190-17. https://doi.org/10.1128/JVI.02190-17.
18. Lucifora, J., Xia, Y., Reisinger, F., Zhang, K., Stadler, D., Cheng, X., Sprinzl, M.F., Koppensteiner, H., Makowska, Z., Volz, T., et al. (2014). Specific and nonhepatotoxic degradation of nuclear hepatitis B virus cccDNA. Science *343*, 1221–1228. https://doi.org/10.1126/science.1243462.
19. Hatakeyama, T., Noguchi, C., Hiraga, N., Mori, N., Tsuge, M., Imamura, M., Takahashi, S., Kawakami, Y., Fujimoto, Y., Ochi, H., et al. (2007). Serum HBV RNA is a predictor of early emergence of the YMDD mutant in patients treated with lamivudine. Hepatology *45*, 1179–1186. https://doi.org/10.1002/hep.21581.
20. Xia, Y., Stadler, D., Lucifora, J., Reisinger, F., Webb, D., Hösel, M., Michler, T., Wisskirchen, K., Cheng, X., Zhang, K., et al. (2016). Interferon-gamma and tumor necrosis factor-alpha produced by T cells reduce the HBV persistence form, cccDNA, without cytolysis. Gastroenterology *150*, 194–205. https://doi.org/10.1053/j.gastro.2015.09.026.
21. Cannataro, V.L., Gaffney, S.G., Sasaki, T., Issaeva, N., Grewal, N.K.S., Grandis, J.R., Yarbrough, W.G., Burtness, B., Anderson, K.S., and Townsend, J.P. (2019). APOBEC-induced mutations and their cancer effect size in head and neck squamous cell carcinoma. Oncogene *38*, 3475–3487. https://doi.org/10.1038/s41388-018-0657-6.
22. Roberts, S.A., Lawrence, M.S., Klimczak, L.J., Grimm, S.A., Fargo, D., Stojanov, P., Kiezun, A., Kryukov, G.V., Carter, S.L., Saksena, G., et al. (2013). An APOBEC cytidine deaminase mutagenesis pattern is widespread in human cancers. Nat. Genet. *45*, 970–976. https://doi.org/10.1038/ng.2702.
23. Hilton, I.B., D'Ippolito, A.M., Vockley, C.M., Thakore, P.I., Crawford, G.E., Reddy, T.E., and Gersbach, C.A. (2015). Epigenome editing by a CRISPR-Cas9-based acetyltransferase activates genes from promoters and enhancers. Nat. Biotechnol. *33*, 510–517. https://doi.org/10.1038/nbt.3199.
24. Dominguez, A.A., Lim, W.A., and Qi, L.S. (2016). Beyond editing: repurposing CRISPR–Cas9 for precision genome regulation and interrogation. Nat. Rev. Mol. Cell Biol. *17*, 5–15.
25. Pickar-Oliver, A., and Gersbach, C.A. (2019). The next generation of CRISPR-Cas technologies and applications. Nat. Rev. Mol. Cell Biol. *20*, 490–507. https://doi.org/10.1038/s41580-019-0131-5.
26. Wang, K., Escobar, M., Li, J., Mahata, B., Goell, J., Shah, S., Cluck, M., and Hilton, I.B. (2022). Systematic comparison of CRISPR-based transcriptional activators uncovers gene-regulatory features of enhancer-promoter interactions. Nucleic Acids Res. *50*, 7842–7855. https://doi.org/10.1093/nar/gkac582.
27. Brezgin, S., Kostyusheva, A., Kostyushev, D., and Chulanov, V. (2019). Dead Cas systems: types, principles, and applications. Int. J. Mol. Sci. *20*, 6041.
28. Colasante, G., Qiu, Y., Massimino, L., Di Berardino, C., Cornford, J.H., Snowball, A., Weston, M., Jones, S.P., Giannelli, S., Lieb, A., et al. (2020). In vivo CRISPRa decreases seizures and rescues cognitive deficits in a rodent model of epilepsy. Brain *143*, 891–905.
29. Gasperini, M., Hill, A.J., McFaline-Figueroa, J.L., Martin, B., Kim, S., Zhang, M.D., Jackson, D., Leith, A., Schreiber, J., Noble, W.S., et al. (2019). A genome-wide framework for mapping gene regulation via cellular genetic screens. Cell *176*, 377–390.e19. https://doi.org/10.1016/j.cell.2018.11.029.
30. Jost, M., Santos, D.A., Saunders, R.A., Horlbeck, M.A., Hawkins, J.S., Scaria, S.M., Norman, T.M., Hussmann, J.A., Liem, C.R., Gross, C.A., and Weissman, J.S. (2020). Titrating gene expression using libraries of systematically attenuated CRISPR guide RNAs. Nat. Biotechnol. *38*, 355–364.
31. Wang, Y., Li, Y., Zai, W., Hu, K., Zhu, Y., Deng, Q., Wu, M., Li, Y., Chen, J., and Yuan, Z. (2022). HBV covalently closed circular DNA minichromosomes in distinct epigenetic transcriptional states differ in their vulnerability to damage. Hepatology *75*, 1275–1288.
32. Zhang, H., Freitas, D., Kim, H.S., Fabijanic, K., Li, Z., Chen, H., Mark, M.T., Molina, H., Martin, A.B., Bojmar, L., et al. (2018). Identification of distinct nanoparticles and subsets of extracellular vesicles by asymmetric flow field-flow fractionation. Nat. Cell Biol. *20*, 332–343.
33. Becirovic, E. (2022). Maybe you can turn me on: CRISPRa-based strategies for therapeutic applications. Cell. Mol. Life Sci. *79*, 130–139.

34. Lackey, L., Law, E.K., Brown, W.L., and Harris, R.S. (2013). Subcellular localization of the APOBEC3 proteins during mitosis and implications for genomic DNA deamination. Cell Cycle *12*, 762–772. https://doi.org/10.4161/cc.23713.

35. Niewiadomska, A.M., Tian, C., Tan, L., Wang, T., Sarkis, P.T.N., and Yu, X.-F. (2007). Differential inhibition of long interspersed element 1 by APOBEC3 does not correlate with high-molecular-mass-complex formation or P-body association. J. Virol. *81*, 9577–9583. https://doi.org/10.1128/JVI.02800-06.

36. Ikeda, T., Abd El Galil, K.H., Tokunaga, K., Maeda, K., Sata, T., Sakaguchi, N., Heidmann, T., and Koito, A. (2011). Intrinsic restriction activity by apolipoprotein B mRNA editing enzyme APOBEC1 against the mobility of autonomous retrotransposons. Nucleic Acids Res. *39*, 5538–5554. https://doi.org/10.1093/nar/gkr124.

37. Chen, Y., Hu, J., Cai, X., Huang, Y., Zhou, X., Tu, Z., Hu, J., Tavis, J.E., Tang, N., Huang, A., and Hu, Y. (2018). APOBEC3B edits HBV DNA and inhibits HBV replication during reverse transcription. Antiviral Res. *149*, 16–25. https://doi.org/10.1016/j.antiviral.2017.11.006.

38. Stadler, D., Kächele, M., Jones, A.N., Hess, J., Urban, C., Schneider, J., Xia, Y., Oswald, A., Nebioglu, F., Bester, R., et al. (2021). Interferon-induced degradation of the persistent hepatitis B virus cccDNA form depends on ISG20. EMBO Rep. *22*, e49568. https://doi.org/10.15252/embr.201949568.

39. Qu, B., Ni, Y., Lempp, F.A., Vondran, F.W.R., and Urban, S. (2018). T5 exonuclease hydrolysis of hepatitis B virus replicative intermediates allows reliable quantification and fast drug efficacy testing of covalently closed circular DNA by PCR. J. Virol. *92*, e01117–e01118.

40. Matharu, N., Rattanasopha, S., Tamura, S., Maliskova, L., Wang, Y., Bernard, A., Hardin, A., Eckalbar, W.L., Vaisse, C., and Ahituv, N. (2019). CRISPR-mediated activation of a promoter or enhancer rescues obesity caused by haploinsufficiency. Science *363*, eaau0629. https://doi.org/10.1126/science.aau0629.

41. Landry, S., Narvaiza, I., Linfesty, D.C., and Weitzman, M.D. (2011). APOBEC3A can activate the DNA damage response and cause cell-cycle arrest. EMBO Rep. *12*, 444–450.

42. Ahodantin, J., Bou-Nader, M., Cordier, C., Mégret, J., Soussan, P., Desdouets, C., and Kremsdorf, D. (2019). Hepatitis B virus X protein promotes DNA damage propagation through disruption of liver polyploidization and enhances hepatocellular carcinoma initiation. Oncogene *38*, 2645–2657.

43. Meyer, B., Voss, K.-O., Tobias, F., Jakob, B., Durante, M., and Taucher-Scholz, G. (2013). Clustered DNA damage induces pan-nuclear H2AX phosphorylation mediated by ATM and DNA–PK. Nucleic Acids Res. *41*, 6109–6118.

44. Qiao, Y., Han, X., Guan, G., Wu, N., Sun, J., Pak, V., and Liang, G. (2016). TGF-beta triggers HBV cccDNA degradation through AID-dependent deamination. FEBS Lett. *590*, 419–427. https://doi.org/10.1002/1873-3468.12058.

45. Faure-Dupuy, S., Riedl, T., Rolland, M., Hizir, Z., Reisinger, F., Neuhaus, K., Schuehle, S., Remouchamps, C., Gillet, N., Schönung, M., et al. (2021). Control of APOBEC3B induction and cccDNA decay by NF-κB and miR-138-5p. JHEP Rep. *3*, 100354. https://doi.org/10.1016/j.jhepr.2021.100354.

46. Petljak, M., Alexandrov, L.B., Brammeld, J.S., Price, S., Wedge, D.C., Grossmann, S., Dawson, K.J., Ju, Y.S., Iorio, F., Tubio, J.M.C., et al. (2019). Characterizing mutational signatures in human cancer cell lines reveals episodic APOBEC mutagenesis. Cell *176*, 1282–1294.e20.

47. Gillmore, J.D., Gane, E., Taubel, J., Kao, J., Fontana, M., Maitland, M.L., Seitzer, J., O' Connell, D., Walsh, K.R., Wood, K., et al. (2021). CRISPR-Cas9 in vivo gene editing for transthyretin amyloidosis. N. Engl. J. Med. *385*, 493–502.

48. Loomba, R., Decaris, M., Li, K.W., Shankaran, M., Mohammed, H., Matthews, M., Richards, L.M., Nguyen, P., Rizo, E., Andrews, B., et al. (2019). Discovery of half-life of circulating hepatitis B surface antigen in patients with chronic hepatitis B infection using heavy water labeling. Clin. Infect. Dis. *69*, 542–545. https://doi.org/10.1093/cid/ciy1100.

49. Fu, Y., Foden, J.A., Khayter, C., Maeder, M.L., Reyon, D., Joung, J.K., and Sander, J.D. (2013). High-frequency off-target mutagenesis induced by CRISPR-Cas nucleases in human cells. Nat Biotech *31*, 822–826.

50. Bogerd, H.P., Kornepati, A.V.R., Marshall, J.B., Kennedy, E.M., and Cullen, B.R. (2015). Specific induction of endogenous viral restriction factors using CRISPR/Cas-derived transcriptional activators. Proc. Natl. Acad. Sci. USA *112*, E7249–E7256. https://doi.org/10.1073/pnas.1516305112.

51. Seeger, C., and Sohn, J.A. (2014). Targeting hepatitis B virus with CRISPR/Cas9. Mol. Ther. Nucleic Acids *3*, e216. https://doi.org/10.1038/mtna.2014.68.

52. Kostyushev, D., Brezgin, S., Kostyusheva, A., Zarifyan, D., Goptar, I., and Chulanov, V. (2019). Orthologous CRISPR/Cas9 systems for specific and efficient degradation of covalently closed circular DNA of hepatitis B virus. Cell. Mol. Life Sci. *76*, 1779–1794. https://doi.org/10.1007/s00018-019-03021-8.

53. Kostyushev, D., Kostyusheva, A., Brezgin, S., Zarifyan, D., Utkina, A., Goptar, I., and Chulanov, V. (2019). Suppressing the NHEJ pathway by DNA-PKcs inhibitor NU7026 prevents degradation of HBV cccDNA cleaved by CRISPR/Cas9. Sci. Rep. *9*, 1847.

54. Kostyushev, D., Kostyusheva, A., Ponomareva, N., Brezgin, S., and Chulanov, V. (2022). CRISPR/Cas and hepatitis B therapy: technological advances and practical barriers. Nucleic Acid Ther. *32*, 14–28.

55. Tropberger, P., Mercier, A., Robinson, M., Zhong, W., Ganem, D.E., and Holdorf, M. (2015). Mapping of histone modifications in episomal HBV cccDNA uncovers an unusual chromatin organization amenable to epigenetic manipulation. Proc. Natl. Acad. Sci. USA *112*, E5715–E5724. https://doi.org/10.1073/pnas.1518090112.

56. Kostyushev, D., Kostyusheva, A., Brezgin, S., Smirnov, V., Volchkova, E., Lukashev, A., and Chulanov, V. (2020). Gene editing by extracellular vesicles. Int. J. Mol. Sci. *21*, 7362.

57. Kostyusheva, A., Brezgin, S., Bayurova, E., Gordeychuk, I., Isaguliants, M., Goptar, I., Urusov, F., Nikiforova, A., Volchkova, E., Kostyushev, D., et al. (2019). ATM and ATR expression potentiates HBV replication and contributes to reactivation of HBV infection upon DNA damage. Viruses *11*, 997. https://doi.org/10.3390/v11110997.

58. Wang, T., Wei, J.J., Sabatini, D.M., and Lander, E.S. (2014). Genetic screens in human cells using the CRISPR-Cas9 system. Science *343*, 80–84. https://doi.org/10.1126/science.1246981.

59. Hart, T., Chandrashekhar, M., Aregger, M., Steinhart, Z., Brown, K.R., MacLeod, G., Mis, M., Zimmermann, M., Fradet-Turcotte, A., Sun, S., et al. (2015). High-Resolution CRISPR screens reveal fitness genes and genotype-specific cancer liabilities. Cell *163*, 1515–1526. https://doi.org/10.1016/j.cell.2015.11.015.

60. Kumar, N., Stanford, W., de Solis, C., Aradhana, Abraham, N.D., Dao, T.M.J., Thaseen, S., Sairavi, A., Gonzalez, C.U., and Ploski, J.E. (2018). The development of an AAV-based CRISPR SaCas9 genome editing system that can Be delivered to neurons in vivo and regulated via doxycycline and cre-recombinase. Front. Mol. Neurosci. *11*, 413. https://doi.org/10.3389/fnmol.2018.00413.

61. Cai, D., Nie, H., Yan, R., Guo, J.-T., Block, T.M., and Guo, H. (2013). A southern blot assay for detection of hepatitis B virus covalently closed circular DNA from cell cultures. Methods Mol. Biol. *1030*, 151–161. https://doi.org/10.1007/978-1-62703-484-5_13.

62. Li, F., Cheng, L., Murphy, C.M., Reszka-Blanco, N.J., Wu, Y., Chi, L., Hu, J., and Su, L. (2016). Minicircle HBV cccDNA with a Gaussia luciferase reporter for investigating HBV cccDNA biology and developing cccDNA-targeting drugs. Sci. Rep. *6*, 36483. https://doi.org/10.1038/srep36483.

63. Alfaiate, D., Dény, P., and Durantel, D. (2015). Hepatitis delta virus: from biological and medical aspects to current and investigational therapeutic options. Antiviral Res. *122*, 112–129. https://doi.org/10.1016/j.antiviral.2015.08.009.

OMTN, Volume *32*

Supplemental information

Transient and tunable CRISPRa regulation of APOBEC/AID genes for targeting hepatitis B virus

Dmitry Kostyushev, Sergey Brezgin, Anastasiya Kostyusheva, Natalia Ponomareva, Ekaterina Bayurova, Natalia Zakirova, Alla Kondrashova, Irina Goptar, Anastasiya Nikiforova, Anna Sudina, Yurii Babin, Ilya Gordeychuk, Alexander Lukashev, Andrey A. Zamyatnin Jr., Alexander Ivanov, and Vladimir Chulanov

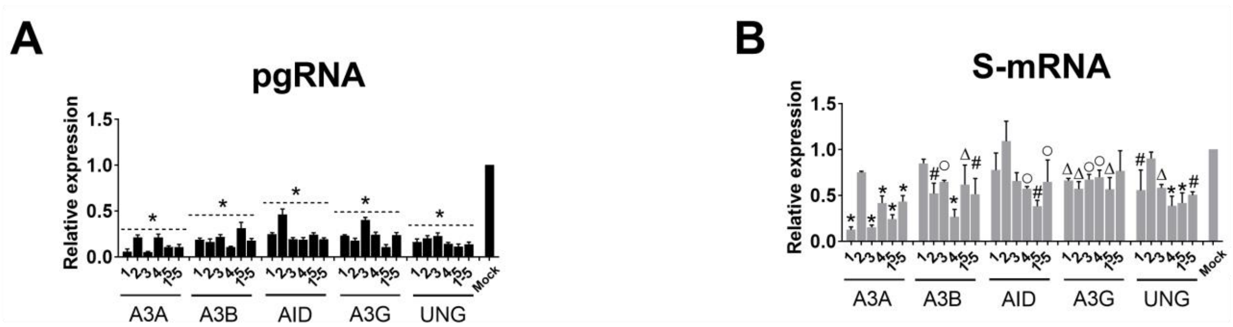

Figure S1. Screening for sgRNAs with highest anti-HBV activity. Anti-HBV activity of sgRNAs targeting A3A, A3B, AID, A3G and UNG genes measured by (A) pgRNA and (B) S-mRNA levels. HBV RNA is provided relative to GAPDH mRNA. $^{\circ}p < 0.05$, $^{\Delta}p < 0.01$, $^{\#}p <0.001$, $^{*}p < 0.0001$. *Mock* – dCas9-p300 transfected with a non-targeting sgRNA. Numbers indicate individual sgRNAs, 1-5 corresponds to all five sgRNAs targeting a single gene used in combination.

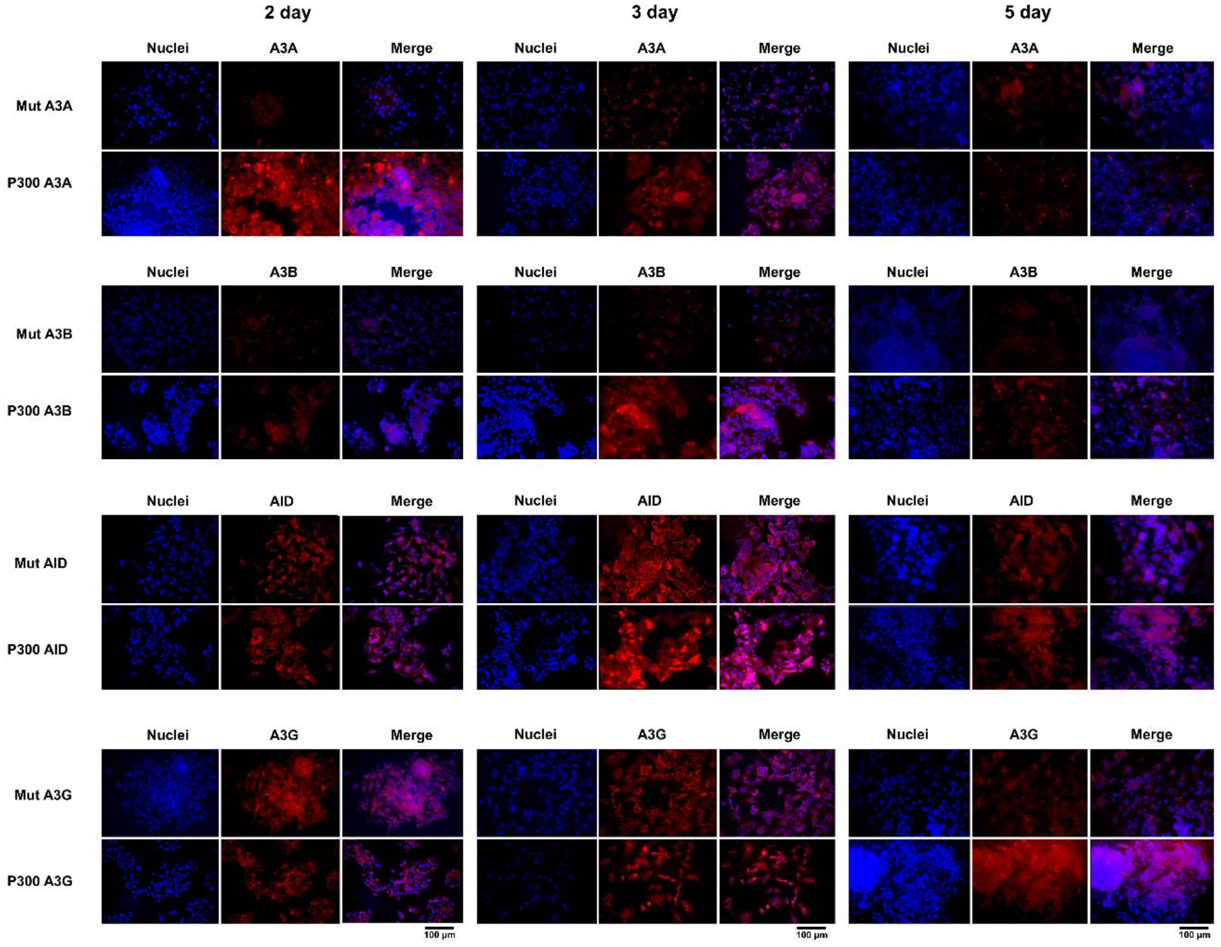

Figure S2. Immunostaining for APOBEC/AID factors. Mut – cells transfected with a mutant form of dCas9-p300 and a targeting sgRNA, P300 – cells transfected with dCas9-p300 and a targeting sgRNA. HepG2 cells were stained for a corresponding APOBEC/AID protein at specified days; cell nuclei are labeled with a Hoechst33342 (blue). Note: these images are used as merged images in Figure 1E.

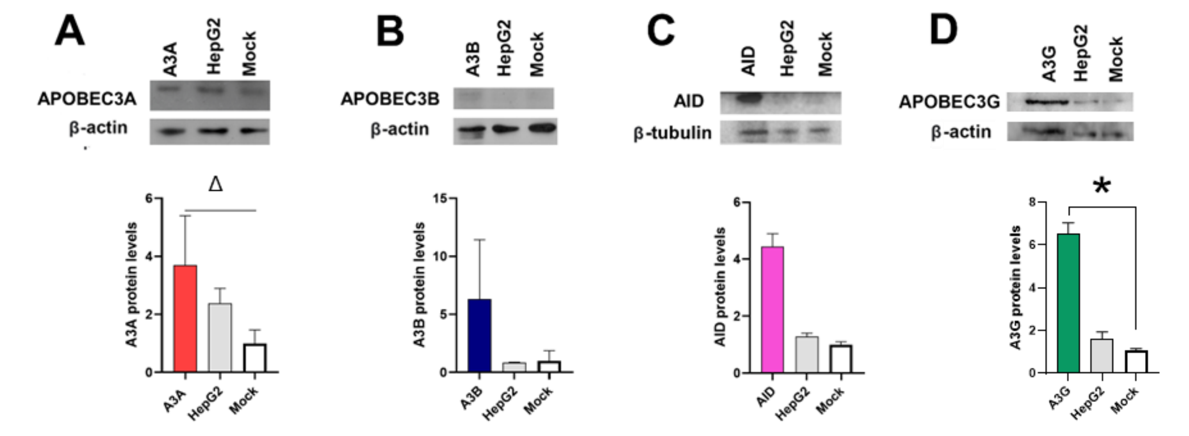

Figure S3. Western blot analysis APOBEC/AID expression. Protein expression of (A) A3A, (B) A3B, (C) AID and (D) A3G. Western blot analysis was performed 42 hours post transfection. Mock, HepG2 cells transfected with dCas9-p300 with a non-targeting sgRNA. Values are expressed as means ± standard deviation of triplicate experiments. HepG2 – not transfected HepG2 cells. $^{\Delta}p < 0.01$, $^{*}p < 0.0001$.

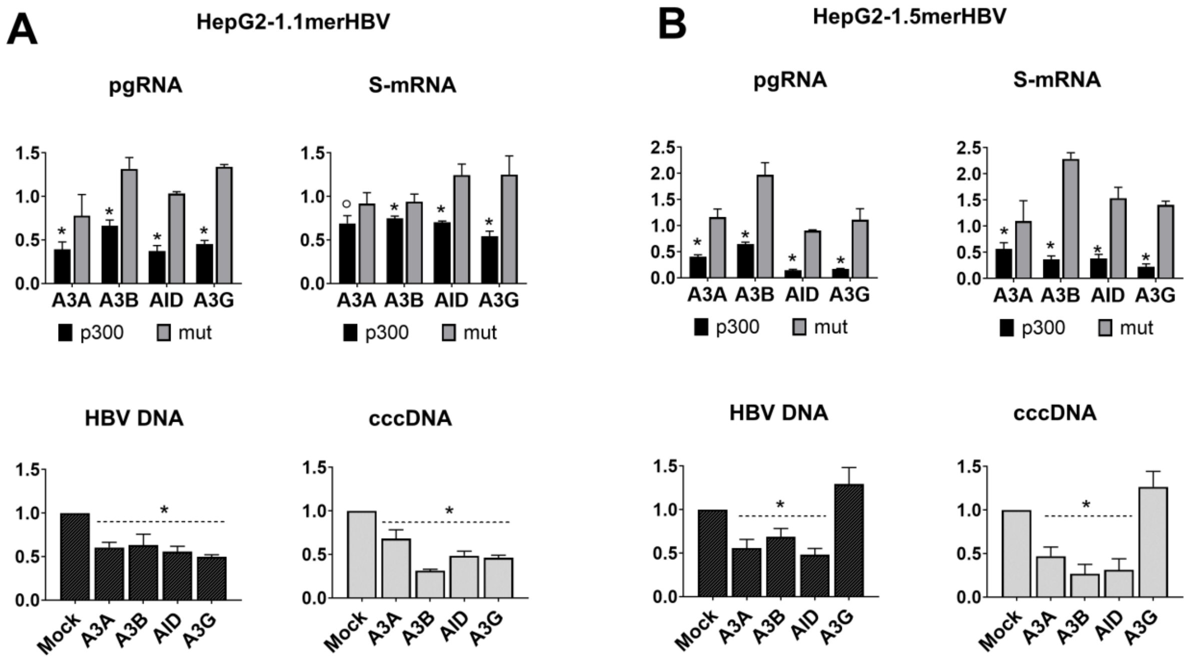

Figure S4. Suppression of HBV replication by CRISPRa at stable cell lines. Anti-HBV activity was analyzed at (A) HepG2-1.1merHBV cells with HBV replication induced from a tet-on CMV promoter de novo by adding doxycycline for 24 hours and (B) HepG2-1.5merHBV cells where HBV is constitutively produced from a wild-type promoter. Anti-HBV activity was measured 5 days post transfection; transfected cells were selected using blasticidin treatment as previously described[1]. HBV DNA and cccDNA are provided relative to genomic β-globin; HBV pgRNA and S-mRNA are relative to GAPDH mRNA. Cell lines were transfected with dCas9-p300 or a mutant form of dCas9-p300 with an sgRNA targeting A3A, A3B, AID or A3G. . $^{\circ}p < 0.05$, $^{\Delta}p < 0.01$, $^{\#}p < 0.001$, $^{*}p < 0.0001$. *Mut* – cells transfected with a mutant form of dCas9-p300 and a

targeting sgRNA, *P300* – cells transfected with dCas9-p300 and a targeting sgRNA. *Mock* – dCas9-p300 transfected with a non-targeting sgRNA.

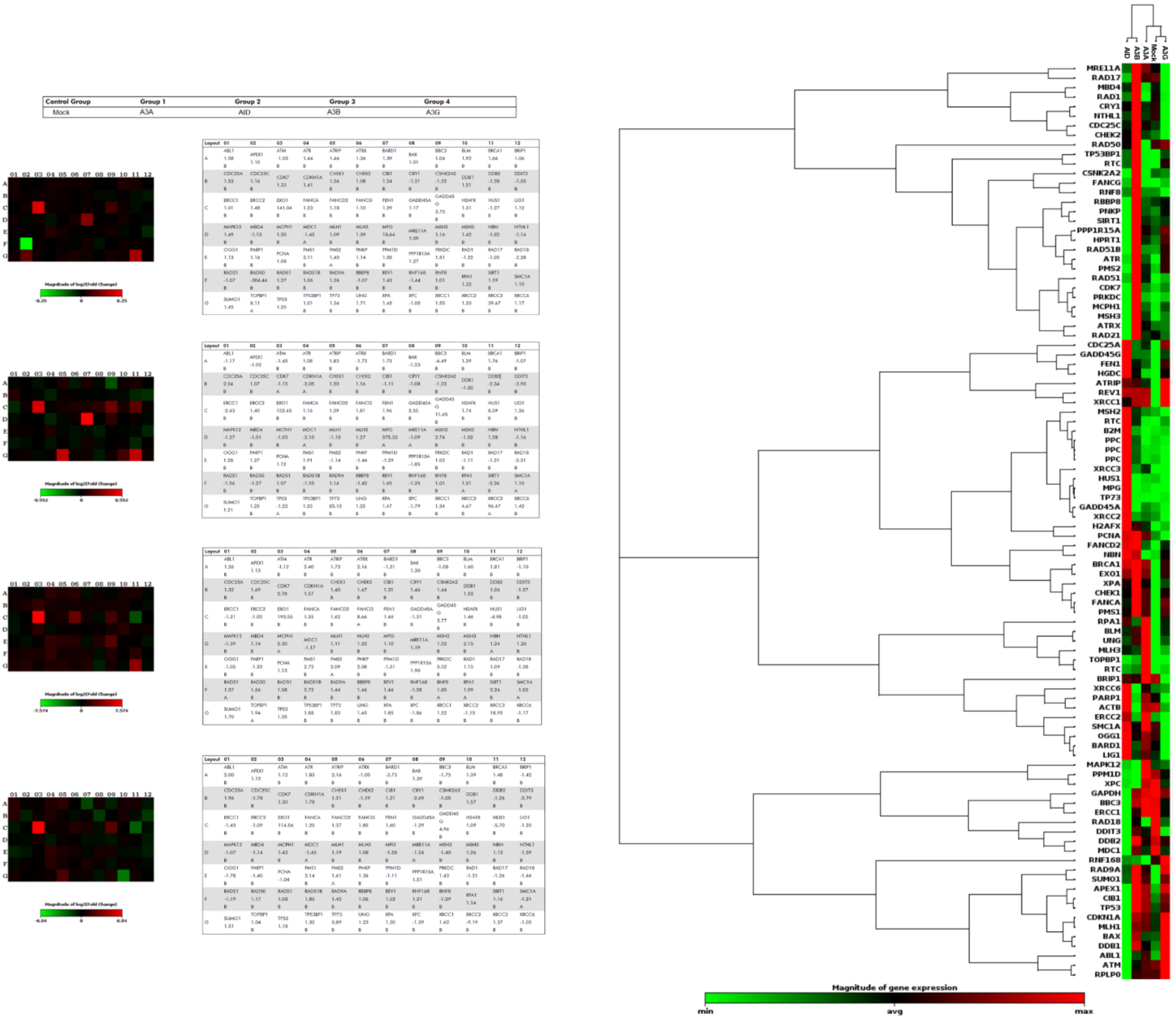

Figure S5. Microarray analysis and clustergram of HepG2-1.1merHBV cells transfected with CRISPRa targeting APOBEC/AID. Cells were transfected with dCas9-p300 targeting A3A, A3B, AID, A3G or with a non-targeting sgRNA (Mock control), and used for microarray profiling 3 days post transfection. Co-regulated genes across groups or individual samples are displayed at a heatmap with dendrograms (right side).

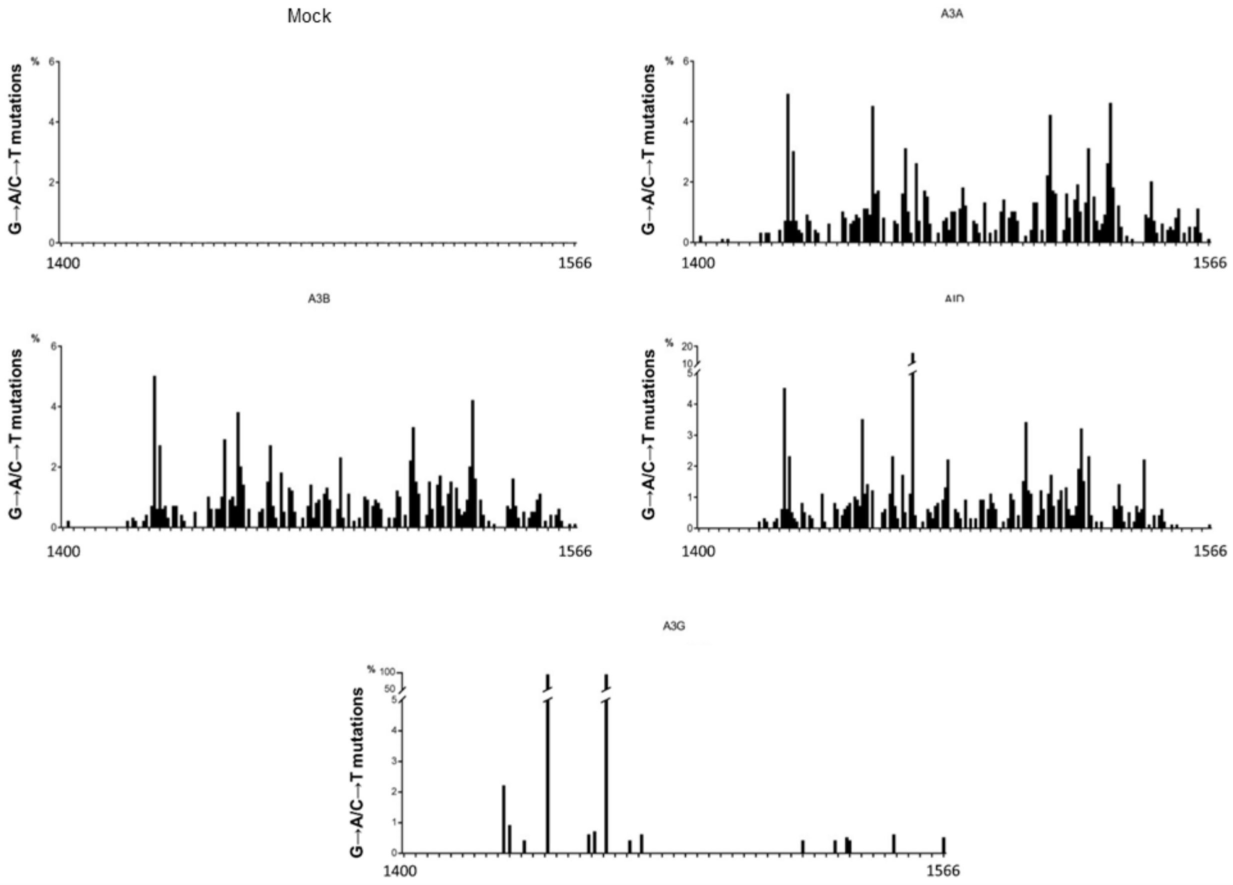

Figure S6. Deamination profile of HBV cccDNA upon APOBEC/AID activation by CRISPRa. HBx region of HBV genome (1400-1566 n.t.) was amplified with specified primers and analyzed by NGS. Deamination map indicates the frequency of G→A/C→T mutations at indicated positions. Mock, dCas9-p300 with a non-targeting sgRNA.

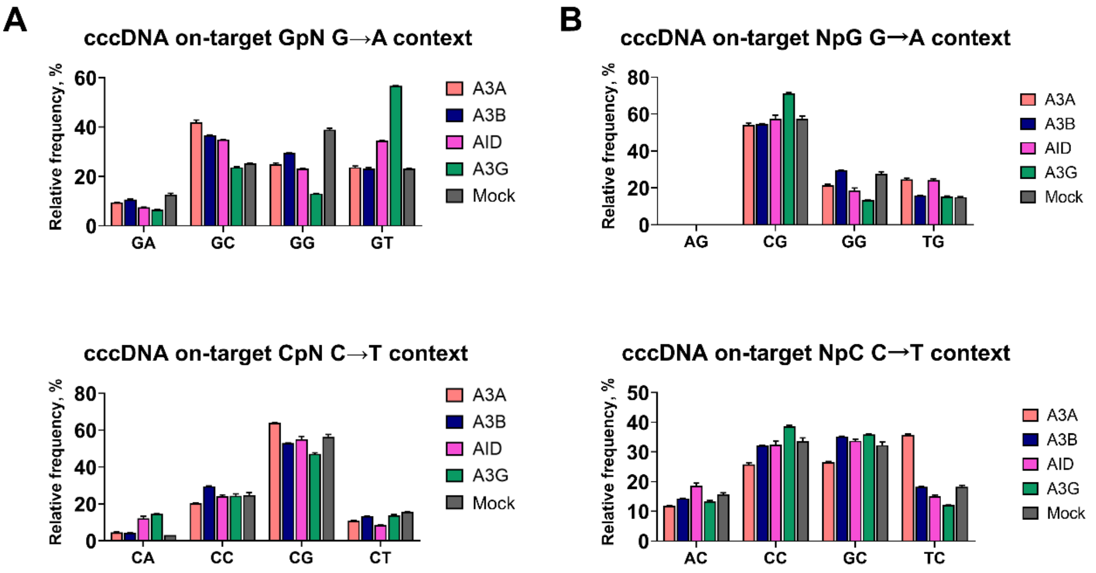

Figure S7. Analysis of cccDNA dinucleotide mutation context. (A) G/CpN context and (B) NpG/C context of G→A and C→T mutations upon CRISPR-activation of A3A, A3B, AID and A3G. Mock – dCas9-p300 system co-transfected with a non-targeting sgRNA. Analysis of NpC/G and C/GpN dinucleotide context showed that upon CRISPRa of A3A cccDNA harbored G→A substitutions mostly in 5’-GC-3’ and 5’-TG-3’ context (underlined base indicates a modified nucleotide), and C→T mutations in 5’-CG-3’ and 5’-TC-3’ context. A3B deaminated cccDNA preferentially in a 5’-GC-3’/5’-GG-3’ and 5’-CC-3’ sites. Frequent 5’-GC-3’/5’-GT-3’ and 5’-TC-3’/5’-AC-3’ mutations were apparent upon AID CRISPRa. A3G showed preference for 5’-GT-3’/5’-CG-3’ and 5’-CA-3’/5’-CC-3’ sites. Mock, dCas9-p300 with a non-targeting sgRNA.

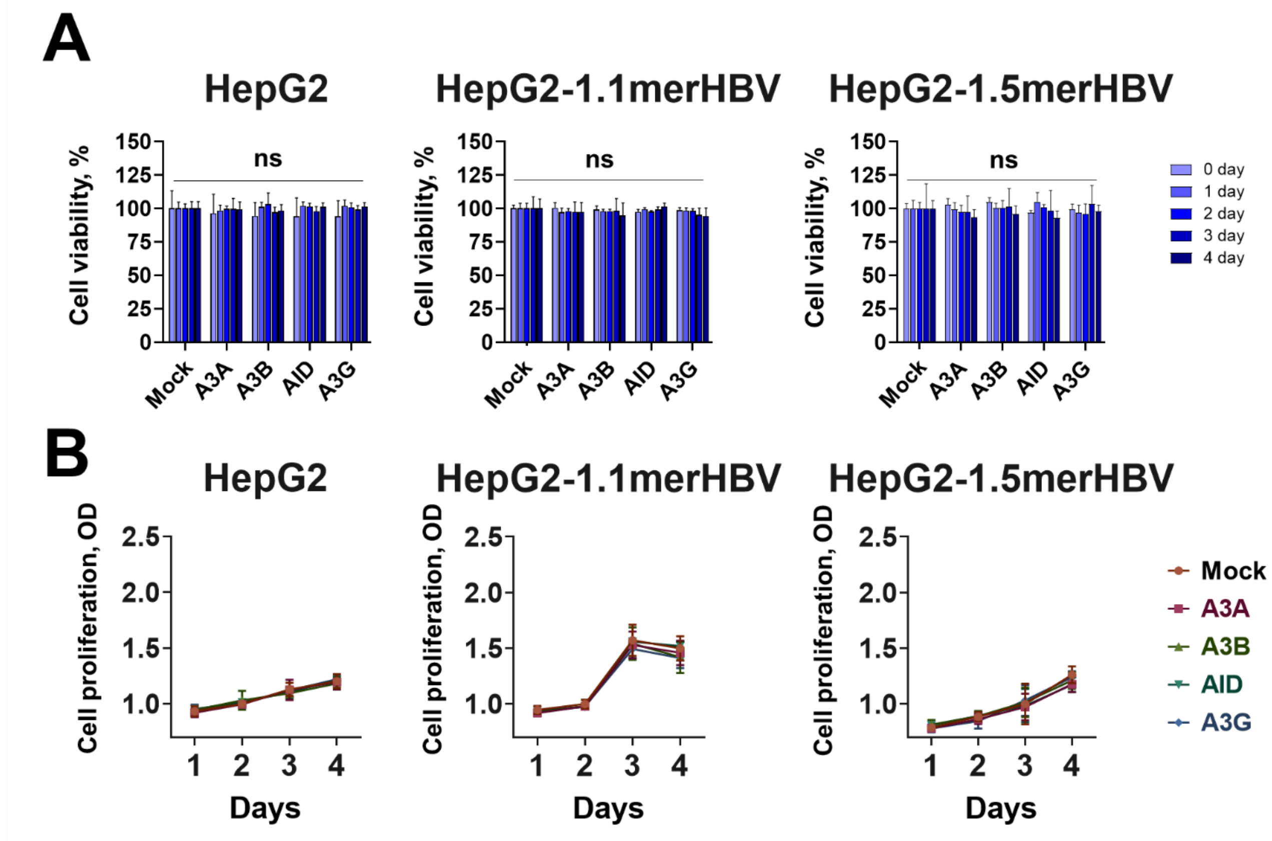

Figure S8. Analysis of APOBEC/AID toxicity. APOBEC/AID genes were activated in HepG2, HepG2-1.1merHBV or HepG2-1.5merHBV cell lines using CRISPRa. Cytotoxicity and proliferation of cells were measured at days 0 to 4 post transfection using Cytotoxicity assay kit (Abcam). Mock, dCas9-p300 with a non-targeting sgRNA. All changes are not statistically significant. *ns* – not significant.

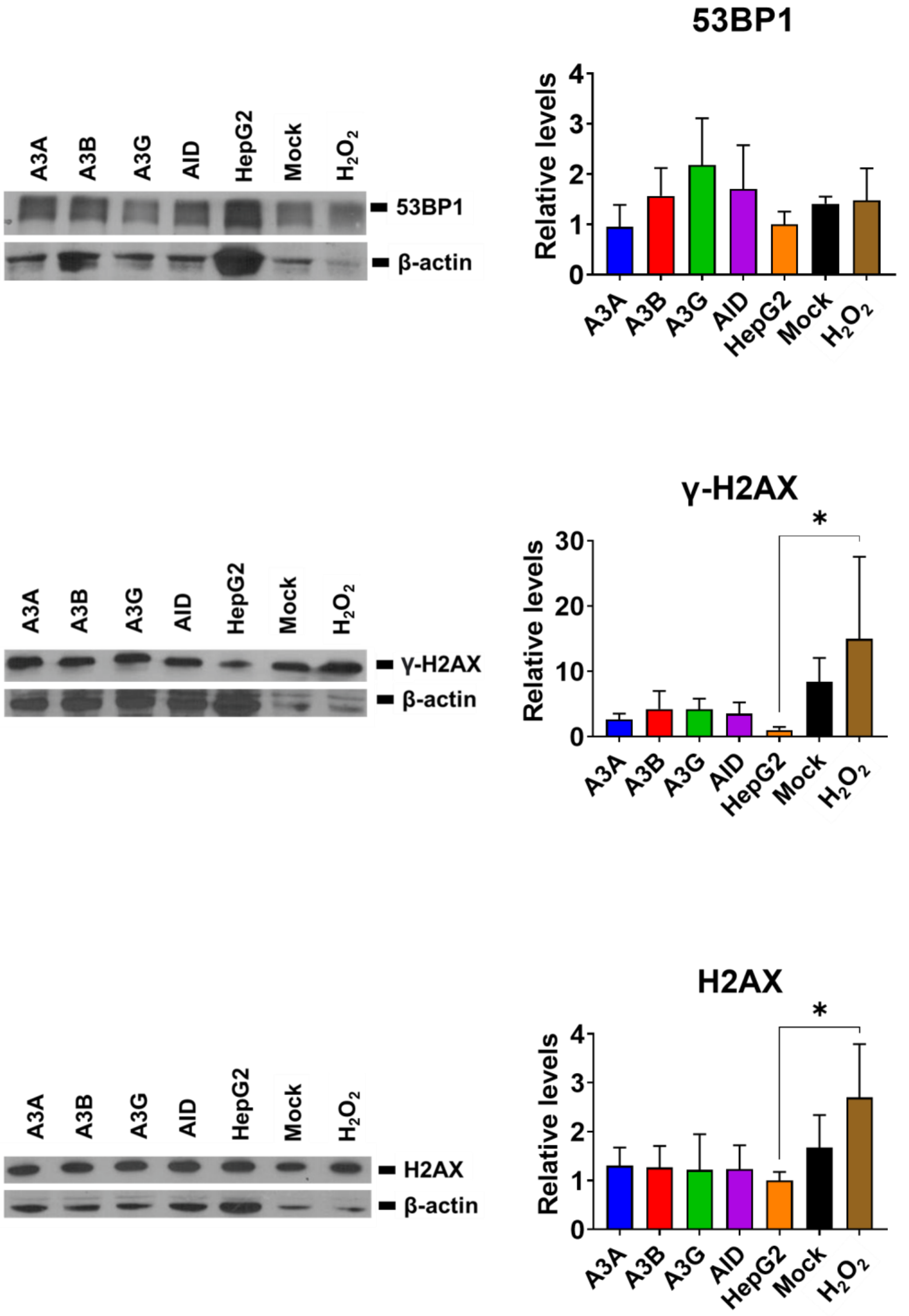

Figure S9. Western blot analysis of 53BP1, γ-H2AX, H2AX, and β-actin in CRISPRa-transfected cells with relative protein densities. Mock, dCas9-p300 with a non-targeting sgRNA.

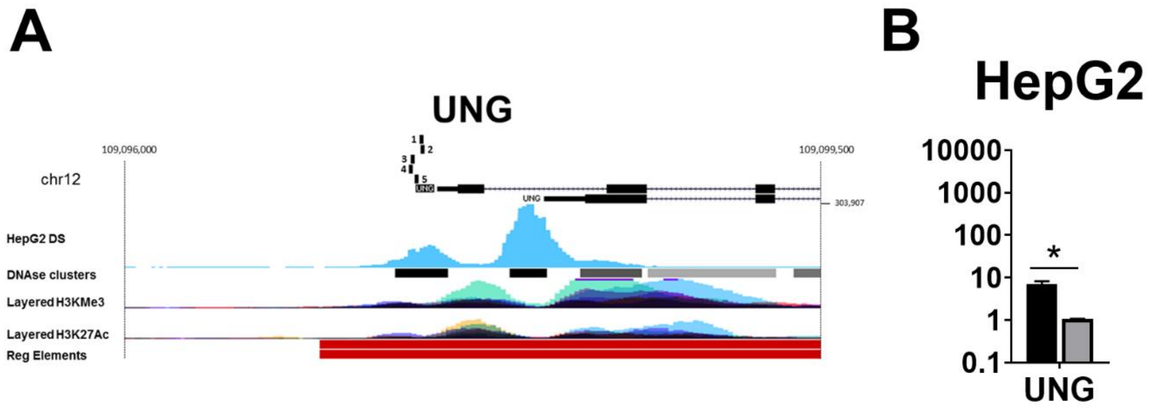

Figure S10. CRISPR-activation of UNG gene. (A) Design of sgRNA targeting UNG promoter region. (B) Peal activation of UNG using CRISPRa. UNG mRNA levels with targeting CRISPRa (black bars) or CRISPRa system with a mutant form of p300 (grey bars). UNG mRNA levels are relative to GAPDH mRNA. $^{\circ}p < 0.05$, $^{\Delta}p < 0.01$, $^{\#}p < 0.001$, $^{*}p < 0.0001$.

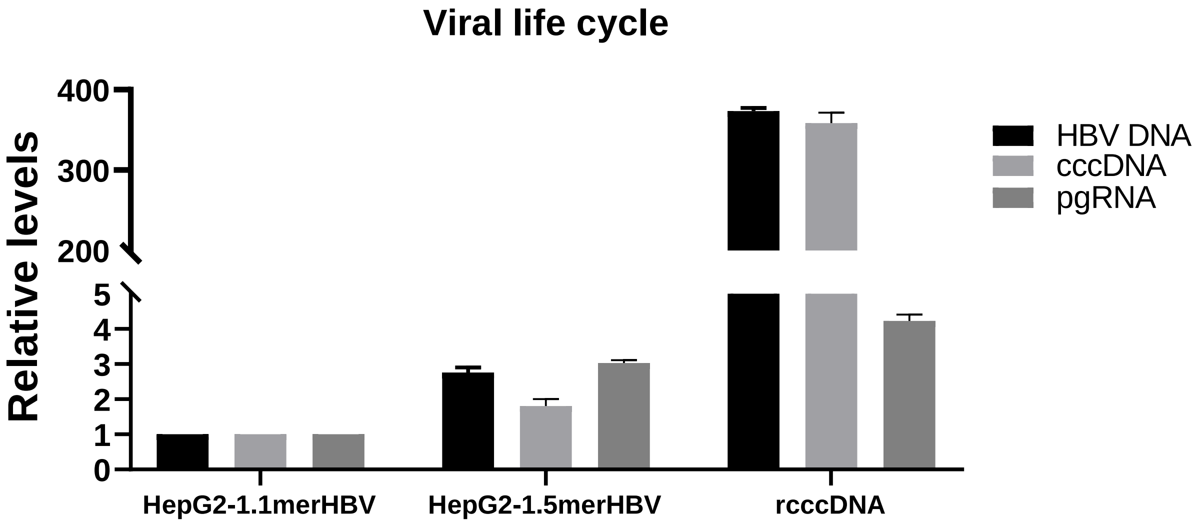

Figure S11. HBV replication at in vitro models. Relative HBV DNA, cccDNA and pgRNA levels were measured at 3 cell lines: HepG2-1.1merHBV with 24 hours of HBV tet-on promoter

activation using doxycycline, HepG2-1.5merHBV with constitutive production of HBV from a wild-type promoter and in HepG2 cells transfected with HBV rcccDNA.

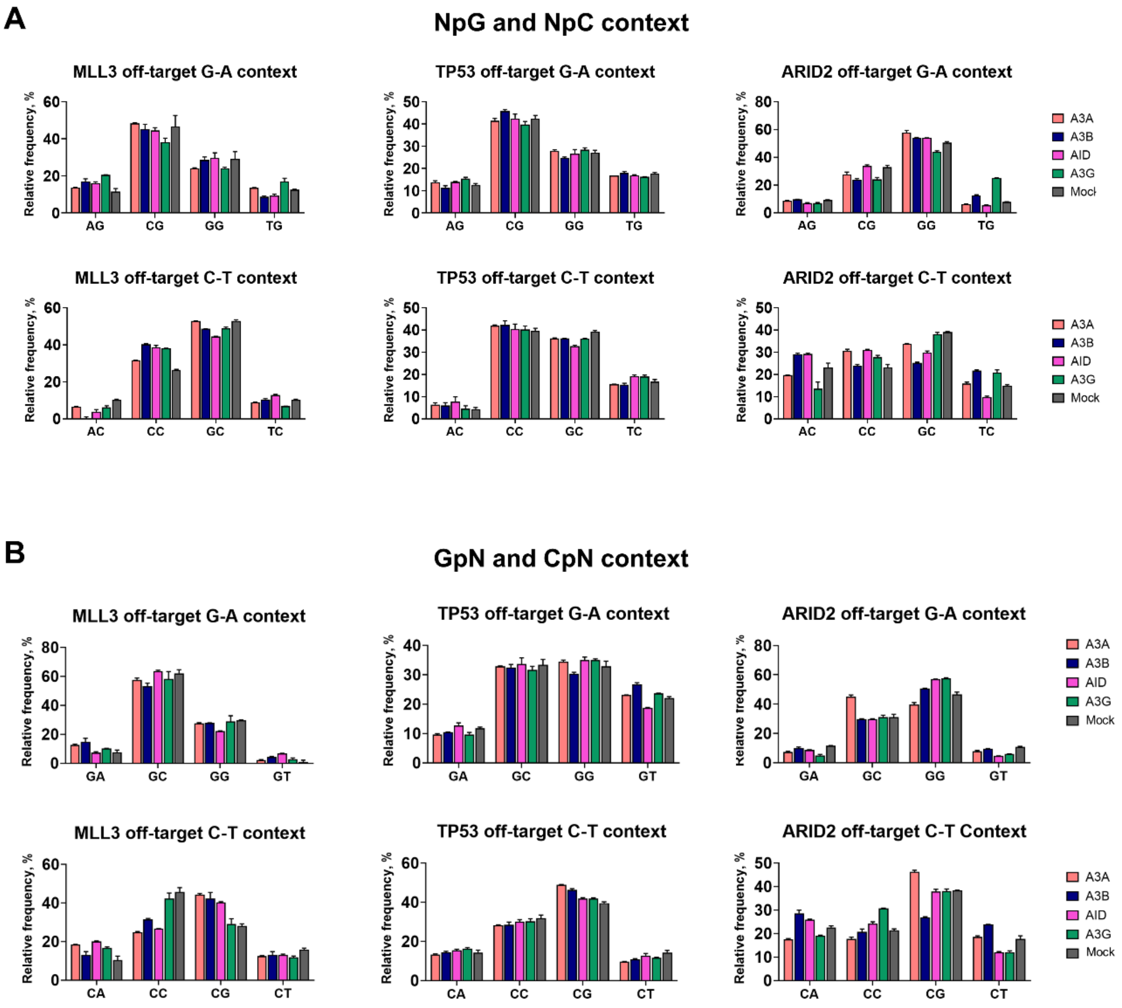

Figure S12. APOBEC/AID mutational context at genomic sites.

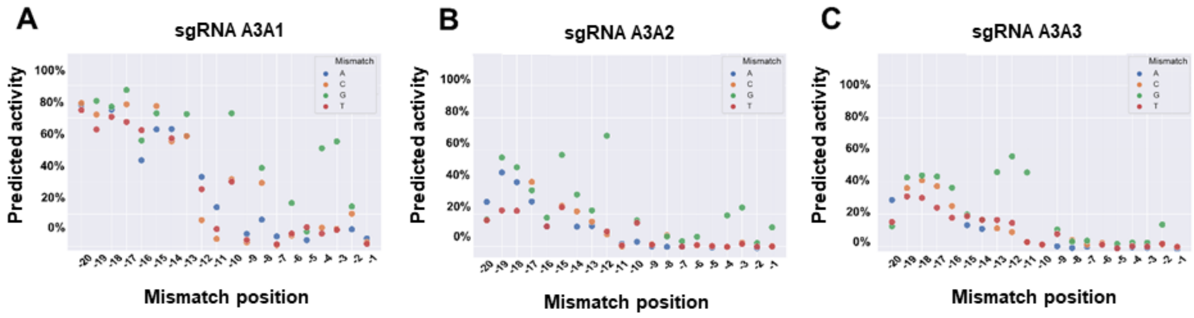

Figure S13. Predicted activity of attenuated sgRNAs for A3A gene.

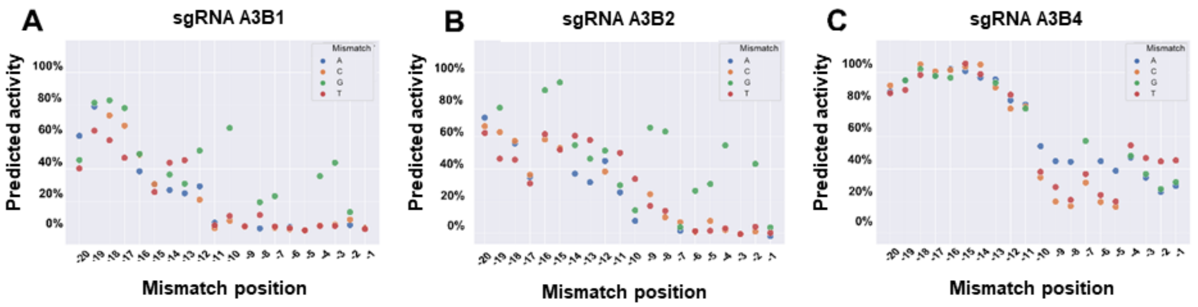

Figure S14. Predicted activity of attenuated sgRNAs for A3B gene.

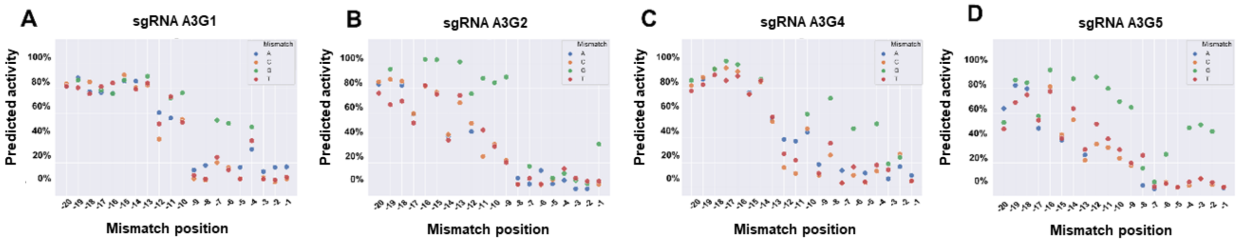

Figure S15. Predicted activity of attenuated sgRNAs for A3G gene.

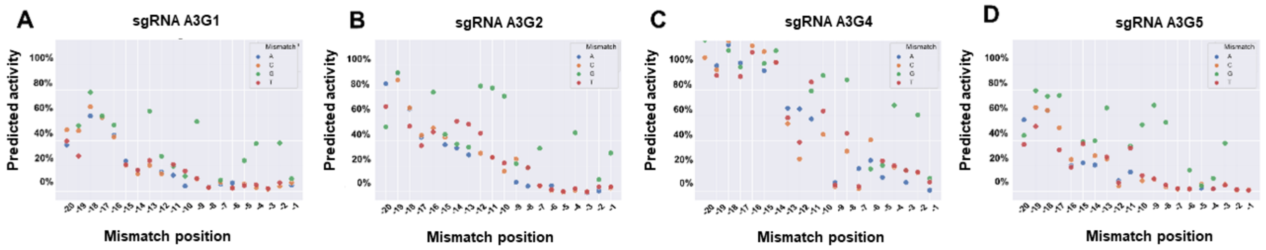

Figure S16. Predicted activity of attenuated sgRNAs for AID gene.

Tables S1-S17 and S20 are supplied as an excel file.

Table S18. Parameters of designed sgRNAs.

Name	5'→3' nucleotide sequence	Predicted efficacy	Chain	MM1	MM2	MM3	MM4	PAM
A3A1	GACACTGTGGAGTGACAATG	0,51	-	0	4	19	113	GGG
A3A2	CCCTCATTAGCGTCAGAACA	0,49	-	0	0	3	25	AGG
A3A3	CAAAACCAAAGCTTGCCCAG	0,7	+	0	2	19	93	AGG
A3A4	GCTAATGAGGGTGGCACACT	0,53	+	0	0	10	86	CGG
A3A5	GGCCCACAGGGAGCAAAGTG	0,65	+	0	2	21	129	TGG
A3B1	CCAATGCCTGAGCAGGAATG	0,81	+	1	5	18	118	GGG
A3B2	GAGGAACCTCCAATAAAGAC	0,55	-	1	0	11	51	AGG
A3B3	ATTGGAGGTTCCTCTGCCAG	0,85	+	0	1	14	77	CGG
A3B4	GCATTGGTGTGGGAGGCCCC	0,79	-	1	11	23	192	GGG
A3B5	CATTGCAGCCTGAGCCTGGG	0,71	-	0	2	33	210	AGG
AID1	ACCCCCACCAGGTACCCCAA	0,71	+	0	2	8	60	CGG
AID2	GATTTGGATCCAGTGAAGAT	0,72	+	0	1	9	71	GGG
AID3	CTGGATCCAAATCAGGAGCA	0,59	-	0	0	12	83	AGG
AID4	TGTCATAGGCAGAGTCACAC	0,66	+	0	0	6	91	AGG
AID5	CATAGCTAGCAAAGATCAGG	0,6	+	0	0	4	75	AGG
A3G1	CCCGCTTCCTGCTCAGGCGC	0,58	-	2	0	4	97	TGG
A3G2	GAGGAAGATAAAGCGTCCCA	0,66	+	0	0	7	32	GGG
A3G3	TAAAGACAGGCCGCTCTGTG	0,77	-	0	1	3	47	CGG
A3G4	AGAAAGAGGGTGAGAGACTG	0,62	+	0	14	116	401	AGG
A3G5	CCCCAGAGAAAACCAGAAAG	0,63	+	0	5	37	228	AGG

Table S19. Primers used for 3D-PCR analysis.

**Name**	**5'→3' nucleotide sequence**
3DcccDNA fw	GCCTATTGATTGGAAAGTATGT
3DcccDNA rev	AGCTGAGGCGGTATCTA
HBxin fw	ATGGCTGCTARGCTGTGCTGCCAA
HBxin rev	AAGTGCACACGGTYYGGCAGAT
